# Allabogdanite, the high-pressure polymorph of (Fe,Ni)_2_P, a stishovite-grade indicator of impact processes in the Fe–Ni–P system

**DOI:** 10.1038/s41598-018-37795-x

**Published:** 2019-01-31

**Authors:** Sergey N. Britvin, Vladimir V. Shilovskikh, Renato Pagano, Natalia S. Vlasenko, Anatoly N. Zaitsev, Maria G. Krzhizhanovskaya, Maksim S. Lozhkin, Andrey A. Zolotarev, Vladislav V. Gurzhiy

**Affiliations:** 1 Institute of Earth Sciences, Saint-Petersburg State University, Universitetskaya Nab. 7/9, 199034 St. Petersburg, Russia; 2 Kola Science Center of Russian Academy of Sciences, Fersman Str. 14, 184209 Apatity, Murmansk Region Russia; 3 Centre for Geo-Environmental Research and Modelling, Saint-Petersburg State University, Ulyanovskaya ul. 1, 198504 St. Petersburg, Russia; 4 Casella Postale 37, Cinisello, Milano Italy; 5 Recource Center “Nanophotonics”, Saint-Petersburg State University, Ulyanovskaya ul. 1, 198504 St. Petersburg, Russia

## Abstract

Allabogdanite, (Fe,Ni)_2_P, is the only known natural high-pressure phase reported in the Fe–Ni–P system. The mineral, which was previously described from a single meteorite, the Onello iron, is now discovered in the Santa Catharina and Barbianello nickel-rich ataxites. The occurrence of allabogdanite in Santa Catharina, one of the largest and well-studied meteorites, suggests that this mineral is more common than was believed. The formation of allabogdanite-bearing phosphide assemblages in a given meteorite provides evidence that it experienced peak pressure of at least 8 GPa at a temperature above 800 °C. Since the pressure-temperature stability parameters of allabogdanite fall within the margins of the stishovite (rutile-type SiO_2_) stability area, the former can be employed as a convenient stishovite-grade indicator of significant impact events experienced by iron and stony-iron meteorites and their parent bodies.

## Introduction

Iron meteorites always attract substantial scientific attention because of their unusual composition and structure^1,2^. Contrary to silicate cosmic material, which is available both from stony meteorites and via delivery by space missions^3^, iron meteorites have no alternative as a source of information on the phase relationships in the metal-rich zones of celestial bodies^2^. Phosphide minerals, i.e. those containing phosphorus in an oxidation state lower than zero, play a significant role in the mineralogy of iron meteorites as a component of the ternary Fe–Ni–P system^4–7^. Iron-nickel phosphides, though accessory minerals, have paramount influence on the crystallization pathways in iron and stony-iron meteorites^8,9^. Natural Fe–Ni phosphides are extensively studied as probable carriers of low-valent phosphorus, which was required for the initiation of the prebiotic phosphorylation processes on the early Earth^10–13^.

One of the main challenges of planetary science is to obtain insights into the evolution of celestial bodies^2^. The records of shock events experienced by planetesimals in their space history are preserved in the high-pressure minerals that are retained in meteorites and impact structures^14^. Unfortunately, the inherent instability of many high-pressure polymorphs under ambient conditions frequently restricts their application to the study of natural objects. In the case of silicate and oxide systems (i.e. stony meteorites and impact craters), the problem is resolved due to a diversity of the high-pressure phases, many of which can be recovered to ambient conditions in the metastable state^15–18^. The situation changes dramatically when we are considering the Fe–Ni–P system: there are just two phases that are stable beyond 5 GPa, but that can be metastably preserved upon quenching. These are (1) a high-pressure synthetic counterpart of the mineral zuktamrurite, FeP_2_^19,20^ and (2) allabogdanite, a high-pressure polymorph of (Fe,Ni)_2_P^21,22^. The practical absence of natural high-pressure indicators in the Fe–Ni–P system restricts the recording of the impact history of iron meteorites to indirect evidence, such as shock-induced shear deformation, twinning (Neumann bands) and impact melting^1,23^ or the extremely rare detection of high-pressure silica polymorphs^24^. Meanwhile, synthetic allabogdanite-type Fe_2_P was shown to be stable from 8 GPa to at least 40 GPa of pressure at a temperature above 800 °C, and can be quenched to the ambient conditions^22,25^. Therefore, allabogdanite, as a natural counterpart of high-pressure Fe_2_P, could serve as a convenient indicator of shock events in the Fe–Ni–P system, having a stability field similar to that of stishovite (high-pressure rutile-type SiO_2_)^22,26^. Like stishovite, which is used as a tracer of shock events in the stony meteorites^27–30^, allabogdanite could indicate significant impact events experienced by the iron and stony-iron meteorites.

Unfortunately, until now, allabogdanite was only known to be present in a single iron meteorite, the nickel-rich ataxite Onello^21^. In the course of ongoing research of the two other nickel-rich ataxites, Santa Catharina and Barbianello, we have confirmed the occurrence of allabogdanite in both of these irons. Herein, we present the results of this current investigation and show that allabogdanite may not be as rare as previously believed, but could often be misidentified as barringerite, a low-pressure polymorph of (Fe,Ni)_2_P^31,32^.

## The Santa Catharina, Barbianello and Onello meteorites

### Santa Catharina

This meteorite was found in 1875 in São Francisco do Sul (Santa Catarina, Brazil)^1^. The total recovered weight of Santa Catharina, at least 7 metric tons, makes it one of the 15 largest meteorites ever found on Earth^1,33^. The meteorite is currently classified as an ungrouped iron related to the IAB complex, and structurally related to nickel-rich ataxites^34^. The latter structural group gathers about 50 iron meteorites having a Ni content exceeding 15 wt.% and featuring a lack of a microscopically resolvable Widmanstätten pattern^1,33^. Santa Catharina was extensively studied due to a chemical similarity between its metal (~35 wt.% of Ni) and the invar-type alloys (ref.^35^ and the citing references). More than 50% of the metal matrix of Santa Catharina is composed of 50-50 iron-nickel metal, having an ordered superstructure of the AuCu (L1_0_) type^36^. The discovery of this mineral, L1_0_-FeNi, now known as tetrataenite^37^, initiated extensive studies resulting in the development of a new class of permanent magnets^38^.

Accessory minerals in Santa Catharina are comprised of phosphides related to the schreibersite-nickelphosphide series, Fe_3_P–Ni_3_P^39,40^, sarcopside-like iron phosphate, Fe_3_(PO_4_)_2_, and magnetite^39,41^. Troilite nodules up to a few centimeters across are very common^41^. Troilite, FeS, is partially substituted by pentlandite, (Ni,Fe)_9_S_8_, and unidentified nickel sulfides^41^. Phosphide mineralization in Santa Catharina was investigated by several scientific groups using electron microprobe^39,41,42^, ion microprobe^43^ and spatially resolved X-ray absorption spectroscopy^44^. Brandstätter and coauthors^42^ reported the phosphide mineral corresponding to (Fe,Ni)_2_P and ascribed it to barringerite, the low-pressure polymorph of (Fe,Ni)_2_P^31^, based on its chemical composition.

### Barbianello

This small (860 g) ungrouped iron meteorite was found in 1961 in Barbianello, Pavia Province, Lombardia, Italy, and described by Fioretti and Zipfel^45^. The metal of Barbianello contains ~27 wt.% of Ni and was severely oxidized by the processes of terrestrial weathering. Phosphides in Barbianello were described as schreibersite^45^, but in fact are represented by its nickel-dominant analogue, nickelphosphide, (Ni,Fe)_3_P^40^.

### Onello

This ungrouped meteorite, found in 1997 in Respublika Sakha (Yakutiya), Russia, is one of the smallest irons ever found (164 g)^46,47^. The nickel content in Onello varies between 22 and 24 wt.%^46^. Phosphides are generally represented by the minerals of the schreibersite-nickelphosphide series, Fe_3_P–Ni_3_P^40,46^. Allabogdanite was discovered in this meteorite in 2002 (ref.^21^) and since that time, it was not known to be anywhere else.

## Results

### Allabogdanite in the Santa Catharina meteorite

**Figure 1:**
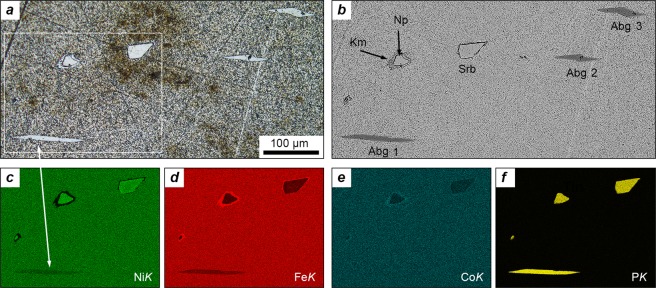
Lamellar allabogdanite crystals and associated phosphides in the metal matrix of the Santa Catharina meteorite. (**a**) Photo in reflected light. (**b**) Image of backscattered electrons (BSE). (**c**–**f**) Elemental maps of the selected region for (**c**) nickel, (**d**) iron, (**e**) cobalt and (**f**) phosphorus. The numbering of allabogdanite crystals corresponds to the analyses numbers given in Table 1. Allabogdanite crystal #1 was used for structure determination. Legend: Abg, allabogdanite; Srb, schreibersite; Np, nickelphosphide; Km, kamacite, α-(Fe,Ni).

The mineral occurs as lamellar crystals up to 120 μm in length and up to 10 μm thick, which are scattered throughout the meteorite matrix. The cross-sections of neighbouring crystals frequently show a non-random, pseudo-collinear orientation (Fig. 1). Allabogdanite is usually associated with the phosphides related to the schreibersite-nickelphosphide series, Fe_3_P–Ni_3_P (Fig. 1). The chemical composition of allabogdanite crystals

(depicted in Fig. 1) is summarized in Table 1. Like allabogdanite from the Onello meteorite^21^, the mineral from Santa Catharina has a uniform chemical composition, shows no signs of chemical zoning and does not interfere with the host Fe-Ni matrix (Fig. 1). Allabogdanite is somewhat enriched in cobalt relative to both Fe-Ni metal and phosphides of the schreibersite-nickelphosphide series (Table 1). The chemical composition and crystal morphology of allabogdanite from Santa Catharina is almost identical to that of barringerite previously described from the same meteorite^42^. It is highly likely that the “barringerite” reported by Brandstätter and co-authors^42^ is in fact allabogdanite, as well.

**Table 1:** Representative chemical composition of allabogdanite and associated minerals.

Mineral	Constituent (wt.%)					Formula	Meteorite
	Fe	Ni	Co	P	Total		
Allabogdanite 1^a^	51.76	24.92	2.10	20.85	99.63	(Fe_1.35_Ni_0.62_Co_0.05_)_2.02_P_0.98_	Santa Catharina
Allabogdanite 2^a^	51.31	25.26	2.32	20.95	99.84	(Fe_1.33_Ni_0.63_Co_0.06_)_2.02_P_0.98_	Santa Catharina
Allabogdanite 3^a^	51.40	25.33	2.20	20.68	99.61	(Fe_1.34_Ni_0.63_Co_0.05_)_2.02_P_0.97_	Santa Catharina
Barringerite^b^	53.7	22.7	2.16	21.6	100.16	(Fe_1.38_Ni_0.56_Co_0.05_)_1.99_P_1.00_	Santa Catharina
Allabogdanite	44.16	32.98	1.10	21.59	99.83	(Fe_1.15_Ni_0.82_Co_0.03_)_2.02_P_1.01_	Barbianello
Allabogdanite^c^	57.65	20.85	2.06	19.33	99.89	(Fe_1.49_Ni_0.48_Co_0.05_)_2.02_P_0.98_	Onello
Schreibersite	44.12	40.17	0.56	14.96	99.81	(Fe_1.61_Ni_1.39_Co_0.02_)_2.02_P_0.98_	Santa Catharina
Nickelphosphide	38.48	46.16	0.39	14.89	99.92	(Ni_1.60_Fe_1.40_Co_0.01_)_2.02_P_0.98_	Santa Catharina
Tetrataenite	52.74	46.62	0.31	bdl^c^	99.67	(Fe_0.54_Ni_0.46_)	Barbianello
Kamacite	92.68	6.15	1.53	bdl	100.36	(Fe_0.93_Ni_0.06_Co_0.01_)	Barbianello
Host metal	66.04	32.77	0.76	bdl	99.57	(Fe_0.67_Ni_0.32_Co_0.01_)	Santa Catharina
Host metal	71.86	27.38	0.94	bdl	100.18	(Fe_0.73_Ni_0.26_Co_0.01_)	Barbianello
Host metal	78.22	20.45	0.91	bdl	99.58	(Fe_0.80_Ni_0.20_Co_0.01_)	Onello

^a^The numbering of analyses corresponds to the allabogdanite crystals numbered in Fig. 1. ^b^The mineral was reported as barringerite based on the chemical composition^42^. ^c^Average of 4 analyses. ^c^bdl: below detection limit.

The crystal structure of allabogdanite from Santa Catharina was determined by an X-ray single-crystal study. It has been solved and refined to *R*_1_ = 0.0455, using a tiny fragment of the crystal #1 depicted in Fig. 1. A brief comparison of the crystallographic data of allabogdanite from the Santa Catharina and Onello meteorites is given in Table 2; the detailed crystallographic data are given in the supporting Crystallographic Information File (CIF). The mineral from Santa Catharina is enriched in nickel relative to the allabogdanite from Onello. The higher nickel content results in noticeable shrinkage of the unit cell axes and, consequently, a reduction of the unit cell volume. The overall effect of Fe for Ni substitution is a 1.4% increase in the density of the mineral in Santa Catharina, relative to that from the Onello meteorite.

**Table 2:** Selected crystallographic data for allabogdanite from the Santa Catharina and Onello meteorites^a^.

	Santa Catharina	Onello
Formula	(Fe_1.33_Ni_0.67_)_2.00_P	(Fe_1.50_Ni_0.50_)_2.00_P
Crystal system	Orthorhombic	Orthorhombic
Space group	*Pnma*	*Pnma*
*a* (Å)	5.7332(7)	5.792(7)
*b* (Å)	3.5413(6)	3.564(4)
*c* (Å)	6.6682(10)	6.691(8)
*V* (Å^3^)	135.38(3)	138.1(3)
*Z*	4	4
*D*_calc_ (g cm^−3^)	7.09	6.93

^a^References: Santa Catharina, this work; Onello, ref.^21^.

### Allabogdanite in the Barbianello meteorite

**Figure 2:**
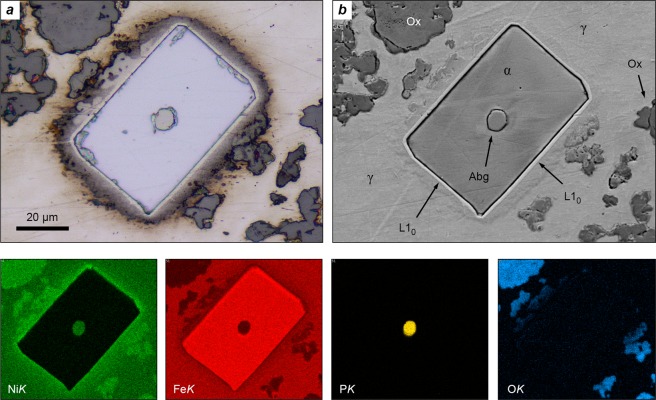
The crystal of allabogdanite residing in the center of the envelope-like kamacite crystal. Kamacite, in due course, is hosted by the taenite single crystal. The thin tetrataenite rim and so-called ≪cloudy zone≫ are visible at the interface between taenite and kamacite. The Barbianello meteorite. (**a**) Photo in reflected light. (**b**) Image of backscattered electrons (BSE). The bottom row shows elemental maps for nickel, iron, phosphorus and oxygen, respectively. Legend (Fig. 2) (**b**): Abg, allabogdanite; α, kamacite [body-centered cubic α-(Fe,Ni)]; γ, taenite [face-centered cubic γ-(Fe,Ni)]; L1_0_, tetrataenite (ordered FeNi); Ox, supergene Fe-Ni oxides.

**Figure 3:**
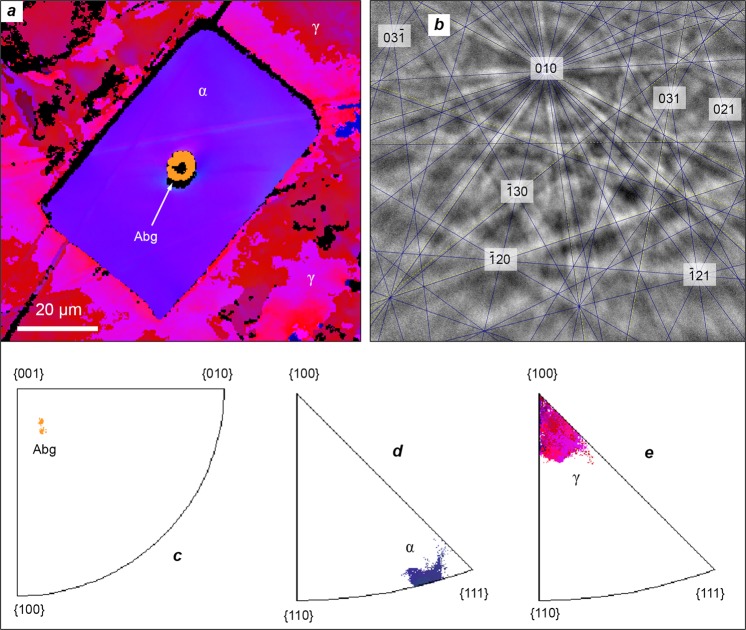
(**a**) Electron backscatter diffraction (EBSD) Euler orientation map of the Barbianello section depicted in Fig. 2. The red-colored host matrix is a slightly deformed taenite single crystal which is cross-cut parallel to its cube face. The blue-colored envelope is a single crystal of kamacite. Allabogdanite crystal (orange-yellow) resides in the center of kamacite crystal. The black regions indicate unindexed areas. (**b**) EBSD pattern from the allabogdanite crystal. The mean angular deviation (MAD) is 0.24° (based on 12 Kikuchi bands). (**c**–**e**) Pole figures showing crystallographic orientation of (**c**) allabogdanite; (**d**) kamacite and (**e**) taenite crystals. Note that [111] axis of kamacite is roughly coincident with [100] axis of taenite. Legend (Fig. 2b): Abg, allabogdanite; α, kamacite [body-centered cubic α-(Fe,Ni)]; γ, taenite [face-centered cubic γ-(Fe,Ni)].

Оnly one crystal of (Fe,Ni)_2_P (Fig. 2) was found in this meteorite, perhaps because of the small available area of the polished section. The size of the allabogdanite crystal (about 7 μm) made the possibility of its safe extraction for the purposes of structure determination doubtful. Therefore, we used the electron backscatter diffraction (EBSD) method for the discrimination between the two possible polymorphs of (Fe,Ni)_2_P: the low-pressure form, barringerite^31^, and the high-pressure polymorph, allabogdanite^21^. The EBSD technique, being inferior to the direct structure solution method, is nevertheless widely used as a non-destructive and highly local method for the purposes of phase diagnostics of micrometer-sized mineral grains^30,48^. The correctness of identification is determined by the value of mean angular deviation (MAD) between the fitted orientation matrices of the model crystal structure and that of the studied mineral phase. MAD values below 1° are considered reliable. In our case, a MAD of 0.24° was obtained for the allabogdanite structure, whereas no acceptable fit was found for the barringerite model. Therefore, EBSD unambiguously signifies that the studied (Fe,Ni)_2_P crystal is allabogdanite (Fig. 3).

Allabogdanite in the Barbianello meteorite is remarkably distinguished from the mineral found in the Santa Catharina and Onello irons. Firstly, it is significantly enriched in nickel (Table 1). Then, contrary to the former meteorites, in which the mineral is hosted by taenite [face-centered cubic γ-(Fe,Ni)], the allabogdanite crystal in the Barbianello meteorite resides in the center of the kamacite [body-centered cubic α-(Fe,Ni)] field (Fig. 2). The latter, in due course, is comprised of a relatively large (50 × 80 μm) single crystal (Fig. 3) rather than an intergrowth of the randomly oriented grains, called “swathing kamacite”^8^. The kamacite crystal is embedded into a single crystal of taenite. The coincidence of [111] axis of kamacite with [100] taenite axis (Fig. 3b,c) suggests a non-random, oriented intergrowth of these two phases. The interface from kamacite towards taenite is traced up by the 1–2 μm tetrataenite rim, followed by a 10-μm thick region of Ni-rich metal known as a “cloudy zone”^37^.

## Discussion

Senateur and coauthors^25^ first reported that the transition of hexagonal Fe_2_P (C22-type, Fe_2_P structure) to the orthorhombic type (C23-type, Co_2_Si structure) occurs at about 8 GPa and 800 °C. The authors, however, did not determine whether the obtained high-pressure phase is stable or metastable under ambient conditions. A further study carried out by Dera and coauthors^22^ substantially expanded the *P-T* stability limits of orthorhombic Fe_2_P, from 8 GPa to 40 GPa and ~1100 °C. The same authors showed that the high-pressure polymorph of Fe_2_P is metastable, but can be retained under the ambient conditions and even re-heated to 1100 °C without back transformation to the low-pressure modification. The mineral allabogdanite, C23-type (Fe,Ni)_2_P, was discovered in the Onello meteorite^21^, where it contains a significant amount of nickel substituting for iron (Table 1). Therefore, allabogdanite, in a strict sense, is not a natural counterpart of Fe_2_P, but rather an intermediate member of (Fe_1-х_Ni_x_)_2_P solid solutions. Because the behaviour of Ni_2_P under high pressure is completely different from that of Fe_2_P^49^, it was not obvious that the pressure-temperature stability limits of pure Fe_2_P could be extrapolated to natural allabogdanite. Although the results reported by Dera and coauthors^22^ ruled out the stabilizing effect of Ni on the allabogdanite-type modification of (Fe,Ni)_2_P, new experimental evidence confirming *P-T* stability limits of the latter phase would be desirable. These experiments have been recently conducted on the Fe–Ni–P system at a pressure of 6 GPa in the temperature range of 900 to 1100 °C; they did not reveal the existence of allabogdanite-type (Fe,Ni)_2_P^50^. Therefore, the lower pressure-temperature stability limit for the high-pressure modification of (Fe,Ni)_2_P lies beyond 6 GPa and 900 °C, in good agreement with the data obtained for pure high-pressure Fe_2_P.

Based on the currently available experimental background, natural allabogdanite can now be considered a high-pressure and high-temperature phase, which can be metastably retained upon quick quenching to ambient conditions. It is noteworthy that the maximum recorded nickel content in allabogdanite from the Onello (0.50 Ni atoms per formula unit, *apfu*), Santa Catharina (0.65 Ni *apfu*) and Barbianello (0.84 Ni *apfu*) meteorites significantly exceeds the allowed Ni content predicted by the theoretical calculations for the high-pressure form of (Fe,Ni)_2_P^51^. Therefore, it is likely that further experiments will shift the upper limit of the pressure-temperature stability area of allabogdanite towards higher *P-T* parameters.

The remarkable similarity between the high-pressure polymorphism of Fe_2_P and silica phases was mentioned by Dera and coauthors^22^. In this respect, allabogdanite can be considered a convenient phosphide counterpart of stishovite in the cosmochemically important Fe–Ni–P system: an indicator of significant shock events experienced by the iron and stony-iron meteorites or their parent bodies. The main obstacle to the application of this mineral as a high-pressure marker is its rarity, because until now, allabogdanite has only been identified in the three iron meteorites mentioned above. Concerning this issue, the discovery stories of allabogdanite in the Onello^21^ and the Santa Catharina meteorites (the present study) are curiously similar. In the Onello meteorite, the mineral was first described as barringerite^46^, the low-pressure polymorph of (Fe,Ni)_2_P^31^, on the basis of its chemical composition. However, the subsequent structural study of “barringerite” from the Onello meteorite resulted in the discovery of allabogdanite^21^. A similar situation occurred with the Santa Catharina mineral, which was formerly reported in this meteorite under the name “barringerite”^42^. The structural determination of “barringerite” from Santa Catharina performed in the present study revealed that this mineral is allabogdanite. It is noteworthy that the Santa Catharina meteorite was extensively studied for more than 30 years. The fact that allabogdanite has been overlooked in one of the best-studied iron meteorites suggests that this mineral might not be as rare as believed.

Among the reported occurrences of meteoritic (Fe,Ni)_2_P (refs^31,52–55^ and the works cited therein), only the first finding (the discovery) of barringerite, the low-pressure form of (Fe,Ni)_2_P, was supported by the X-ray diffraction data^31^. Herein, we showed that allabogdanite may frequently be misidentified as barringerite on the basis of only chemical (electron microprobe) data, leading to the eventual loss of important information related to the impact history of the studied meteorites. Fortunately, the two polymorphs of (Fe,Ni)_2_P, low-pressure barringerite and high-pressure allabogdanite, can be readily distinguished by the electron backscatter diffraction (EBSD) technique. The latter method, being non-destructive, very local and readily accessible, could facilitate the correct identification of allabogdanite, allowing us to record the impact history of meteorites, their parent bodies and possibly, the impact events that occurred on the Earth.

The occurrence of allabogdanite in nickel-rich ataxites opens new insights into the origin and space history of this meteorite group. The stability field of the high-pressure modification of (Fe,Ni)_2_P leads to two reliable scenarios of the formation of nickel-rich ataxites. The first scenario implies equilibrium crystallization of allabogdanite inside the Fe,Ni-metal-rich parts of a large celestial body under a pressure exceeding 8 GPa, which corresponds to the conditions of the Earth’s upper mantle at a depth greater than 250 km^56^. Subsequent fragmentation of the allabogdanite-bearing zone and quenching of the produced fragments could be accomplished through catastrophic collision of the allabogdanite parent body and the external impactor. In this case, nickel-rich ataxites represent examples of impact ejecta, which preserve information about the composition of the inner zone of their parent body.

The second scenario is consistent with the currently accepted formation scheme for the shocked (impact-veined) ordinary chondrites^15–18,30^. In this case, the primary precursor of allabogdanite was its low-pressure polymorph, barringerite^31^, which underwent crystal-to-crystal (topotactic) phase transformation^22^ during collision of the two metal-rich planetesimals. The pressure-temperature conditions of such impact events^15–18^ fall within the margins of the allabogdanite stability field. Besides the presence of allabogdanite, the studied meteorites exhibit common fracturing of the schreibersite-nickelphosphide crystals and brecciated structures of the troilite nodules (in the Santa Catharina meteorite), which supports the hypothesis of the experienced dynamic shock event.

From the authors’ point of view, the second scenario of the formation of nickel-rich ataxites seems more reliable because it does not require the existence of a large planetary body. However, some evidence, such as the crystallographically coincident growth of kamacite and taenite single crystals, as well as the diffusion-driven formation of the “cloudy zone” in the Barbianello meteorite (Fig. 2) argues for the long-time growth of the host Fe,Ni metal under equilibrium conditions^5,6,8,9,57^. It is important, however, that irrespective of the scenario realized, the Fe,Ni metal of the nickel-rich ataxites experienced rapid quenching in the solid state *after* the formation of allabogdanite, and was never reheated to a high temperature (800–1100 °C)^22,25^ upon the pressure release. Otherwise, in spite of known kinetic barriers of the allabogdanite-barringerite transition, the high-pressure form (allabogdanite) could not be preserved in the meteorite fragments. The latter means, in due course, that the cooling history of nickel-rich ataxites is principally different from the thermal history of the common magmatic groups of iron meteorites, which are known to have cooling rates of about 100–10000 °C/Myr^2,9,57^.

## Conclusion

In the present study, we discovered allabogdanite, a high-pressure polymorph of (Fe,Ni)_2_P, in two iron meteorites, Santa Catharina and Barbianello. It is shown that allabogdanite is not a mineralogical curiosity and can be discovered in other meteorites upon detailed study. The mineral is routinely misidentified for its low-pressure analogue, barringerite, but can be readily distinguished from the latter using the non-destructive EBSD (electron backscatter diffraction) method. Allabogdanite, being the only known high-pressure mineral in the Fe–Ni–P system, can be used as a convenient stishovite-grade indicator of significant impact events experienced by the iron (and possibly other classes of) meteorites and their parent bodies.

## Materials and Methods

### Meteorites

Two specimens of the Santa Catharina meteorite (10 and 28 g weight) were obtained from the collection of Sergey Vasiliev (Prague, Czech Republic; svmeteorites.com). The bar-like section of the Barbianello meteorite, 2 × 2 × 20 mm, was kindly loaned by R.P. from his collection. The specimen of the Onello iron, about 6 g weight, is the same one which has been described in the primary reference devoted to allabogdanite^21^.

### Sample preparation

The specimens of the Santa Catharina and Onello meteorites were cut into a few slices; the specimen of the Barbianello meteorite was used as received. The cut sections were polished using conventional metallographic procedures and slightly etched with the nital etchant, in order to develop the phosphide inclusions.

### Electron microprobe study

Electron microprobe analyses (EMPA) of phosphides and the metal were carried out using uncoated samples in energy dispersive mode (acceleration voltage 20 kV, beam current 2 nA), by means of a Hitachi S-3400N scanning electron microscope equipped with an Oxford Instruments AzTec Energy X-Max 20 spectrometer; acquisition time was set to 30 s per point. The following analytical standards were used: InP (P*K*), metallic Fe (Fe*K*), Co (Co*K*) and Ni (Ni*K*). No other elements with the atomic number greater than 4 were detected.

### X-ray single crystal studies

The crystals of phosphides intended for the X-ray single crystal examination were extracted from the appropriate Santa Catharina sections after the local etching of the metal matrix in warm 15% HCl. Several crystal fragments of allabogdanite were extracted and checked with respect to suitability for the single-crystal study. The crystal #1 fragment (Fig. 1) was found to have the best quality. Single-crystal data were collected by means of a Bruker Kappa APEX DUO diffractometer equipped with a flat APEX II CCD detector, using Mo*K*α radiation generated by the microfocus tube. Data collection was performed using Bruker Apex2 software^58^; subsequent data processing and integration routines were conducted using CrysAlis PRO program^59^. Crystal structure of allabogdanite from the Santa Catharina meteorite was solved and refined by means of a *SHELX*-2014 suite of programs^60^ embedded into Olex2 program package^61^. Supplementary Tables 1–3 and crystallographic information file (CIF) in the Supplementary Information contain the details of data collection, structure solution and refinement, atomic coordinates and thermal displacement parameters for allabogdanite from the Santa Catharina meteorite.

### Electron backscatter diffraction (EBSD)

Polished sections suitable for the EBSD study were prepared using reactive ion etching (RIE) with Ar^+^ ions, by means of an Oxford Instruments IonFab-300 instrument operated at 500 V acceleration voltage and 2.4 mA/cm^2^ current density. EBSD measurements were carried out by means of a Hitachi S-3400N scanning electron microscope equipped with an Oxford Instruments Nordlys-HKL EBSD detector, operated at 20 kV and 1.5 nA in focused beam mode with a 70 ° tilted stage. The structures of the minerals were determined by matching the respective EBSD patterns with the reference structural models^21,62,63^.

## Electronic supplementary material

Allabogdanite_CIF

Supplementary Information

## Data Availability

The analytical and crystallographic data are included in this published article (and its Supplementary Information files) but also are available from the corresponding author on reasonable request. Crystallographic data for allabogdanite from the Santa Catharina meteorite have been deposited at the Cambridge Crystallographic Data Center (deposition number CSD 1869223). These data can be obtained free of charge from the Cambridge Crystallographic Data Center *via* www.ccdc.cam.ac.uk/data_request/cif.

## References

[1] Buchwald, V. F. *Handbook of Iron Meteorites, their History, Distribution, Composition and Structure*. University of California Press, Los Angeles, CA (1975).

[2] Goldstein JI, Scott ERD, Chabot NL (2009). Iron meteorites: Crystallization, thermal history, parent bodies, and origin. Chem. Erde – Geochem..

[3] Westphal, A. J. *et al*. The future of Stardust science. *Meteorit. Planet. Sci*. 1–40 (2017).

[4] Vogel R, Baur H (1931). Über das ternäre System Eisen‐Nickel‐Phosphor. Arch. Eisenhuttenwes..

[5] Doan AS, Goldstein JI (1970). The ternary phase diagram, Fe–Ni–P. Metall. Trans..

[6] Romig AD, Goldstein JI (1981). Low temperature phase equilibria in the Fe–Ni and Fe–Ni–P systems: application to the thermal history of metallic phases in meteorites. Geochim. Cosmochim. Acta.

[7] Miettinen J, Vassilev-Urumov G (2015). Thermodynamic description of ternary Fe-X-P systems. Part 6: Fe-Ni-P. J. Phase Equilib. Diff..

[8] Goldstein JI, Doan AS (1972). The effect of phosphorus on the formation of the Widmanstätten pattern in iron meteorites. Geochim. Cosmochim. Acta.

[9] Goldstein JI, Yang J, Scott ERD (2014). Determining cooling rates of iron and stony-iron meteorites from measurements of Ni and Co at kamacite-taenite interfaces. Geochim. Cosmochim. Acta.

[10] Bryant DE (2013). Hydrothermal modification of the Sikhote-Alin iron meteorite under low pH geothermal environments. A plausibly prebiotic route to activated phosphorus on the early Earth. Geochim. Cosmochim. Acta.

[11] Pirim C (2014). Investigation of schreibersite and intrinsic oxidation products from Sikhote-Alin, Seymchan, and Odessa meteorites and Fe_3_P and Fe_2_NiP synthetic surrogates. Geochim. Cosmochim. Acta.

[12] Britvin SN, Murashko MN, Vapnik Y, Polekhovsky YS, Krivovichev SV (2015). Earth’s phosphides in Levant and insights into the source of Archaean prebiotc phosphorus. Sci. Rep..

[13] Pasek MA, Gull M, Herschy B (2017). Phosphorylation on the early earth. Chem. Geol..

[14] French BM, Koeberl C (2010). The convincing identification of terrestrial meteorite impact structures: What works, what doesn’t, and why. Earth Sci. Rev..

[15] Stöffler D, Keil K, Scott ERD (1991). Shock metamorphism of ordinary chondrites. Geochim. Cosmochim. Acta.

[16] Gillet P, El Goresy A (2013). Shock events in the solar system: the message from minerals in terrestrial planets and asteroids. Annual Rev. Earth Planet. Sci..

[17] Tomioka N, Miyahara M (2017). High-pressure minerals in shocked meteorites. Meteorit. Planet. Sci..

[18] Stöffler D, Hamann C, Metzler K (2018). Shock metamorphism of planetary silicate rocks and sediments: Proposal for an updated classification system. Meteorit. Planet. Sci..

[19] Britvin, S. N. *et al*. Zuktamrurite, FeP_2_, a new mineral, the phosphide analogue of löllingite, FeAs_2_. *Phys. Chem. Minerals*, 10.1007/s00269-018-1008-4 (2018).

[20] Gu T-T (2012). High-pressure and high-temperature *in situ* X-ray diffraction study of FeP_2_ up to 70 GPa. Chinese Phys. Lett..

[21] Britvin SN, Rudashevsky NS, Krivovichev SV, Burns PC, Polekhovsky YS (2002). Allabogdanite, (Fe,Ni)_2_P, a new mineral from the Onello meteorite: the occurrence and crystal structure. Am. Mineral..

[22] Dera P (2008). High-pressure polymorphism of Fe_2_P and its implications for meteorites and Earth’s core. Geophys. Res. Lett..

[23] Yang J (2011). Thermal and impact histories of reheated group IVA, IVB, and ungrouped iron meteorites and their parent asteroids. Meteorit. Planet. Sci..

[24] Holtstam D, Broman C, Söderhielm J, Zetterqvist A (2003). First discovery of stishovite in an iron meteorite. Meteorit. Planet. Sci..

[25] Senateur JP, Rouault A, Fruchart R (1976). Etude par spectrometrie Mossbauer des transformations cristallographiques sous hautes pressions de MnFeAs et Fe_2_P. Mater. Res. Bull..

[26] Kuwayama Y (2008). Ultrahigh pressure and high temperature experiments using a laser heated diamond anvil cell in multimegabar pressures region. Rev. High Pres. Sci. Tech..

[27] El Goresy A, Dubrovinsky L, Sharp TG, Chen M (2004). Stishovite and post-stishovite polymorphs of silica in the shergotty meteorite: their nature, petrographic settings versus theoretical predictions and relevance to Earth’s mantle. J. Phys. Chem. Solids.

[28] Miyahara M (2014). Discovery of coesite and stishovite in eucrite. P. Natl. Acad. Sci..

[29] Pang R-L, Zhang A-C, Wang S-Z, Wang R-C, Yurimoto H (2016). High-pressure minerals in eucrite suggest a small source crater on Vesta. Sci. Rep..

[30] Baziotis I (2018). High pressure minerals in the Château-Renard (L6) ordinary chondrite: implications for collisions on its parent body. Sci. Rep..

[31] Buseck PR (1969). Phosphide from meteorites: Barringerite, a new iron-nickel mineral. Science.

[32] Britvin SN, Murashko MN, Vapnik E, Polekhovsky YS, Krivovichev SV (2017). Barringerite Fe_2_P from pyrometamorphic rocks of the Hatrurim Formation, Israel. Geol. Ore Deposits.

[33] Grady, M. M. Catalogue of meteorites, 5th edition. London: The Natural History Museum (2000).

[34] Wasson JT, Kallemeyn GW (2002). The IAB iron-meteorite complex: A group, five subgroups, numerous grouplets, closely related, mainly formed by crystal segregation in rapidly cooling melts. Geochim. Cosmochim. Acta.

[35] Danon J, Scorzelli RB, Souza-Azevedo I, Laugier J, Chamberod A (1980). Santa Catharina meteorite and phase composition of irradiated iron-nickel Invar alloys. Nature.

[36] Danon J (1979). Iron-nickel 50-50 superstructure in the Santa Catharina meteorite. Nature.

[37] Clarke RS, Scott ERD (1980). Tetrataenite – ordered FeNi, a new mineral in meteorites. Am. Mineral..

[38] Makino A (2015). Artificially produced rare-earth free cosmic magnet. Sci. Rep..

[39] Zhang, J., Williams, D. B., Goldstein, J. I. & Clarke, R. S. jr. Electron microscopy study of the iron meteorite Santa Catharina. *Meteoritics* **25**, 167–175 (1990).

[40] Britvin SN (1999). Nickelphosphide (Ni,Fe)_3_P – the nickel analog of schreibersite. Proc. Russ. Mineral. Soc..

[41] Van Tassel R, Dillen H, Vochten R, Degrave E, Hertogen J (1992). An overlooked fragment of the Santa Catharina ataxite. Meteoritics.

[42] Brandstätter, F., Nazarov, M. A. & Kurat, G. Barringerite from the Santa Catharina ungrouped iron meteorite. *Abstracts 34th Annual Lunar and Planetary Science Conference*, Abstr. 1681, https://www.lpi.usra.edu/meetings/lpsc2003/pdf/1681.pdf (2003).

[43] Miller MK, Russell KF (1992). An APFIM investigation of a weathered region of the Santa Catharina meteorite. Surf. Sci..

[44] Schofield PF (2010). X-ray spectromicroscopy of mineral intergrowths in the Santa Catharina meteorite. Geostand. Geoanal. Res..

[45] Fioretti AM, Zipfel J (2004). Barbianello: An ungrouped nickel-rich iron meteorite found in Italy. Meteorit. Planet. Sci..

[46] Kopylova AG, Oleinikov BV, Sobolev NV, Sushko OA (1999). A new iron meteorite Onello – unique nickel-rich ataxite. Dokl. Akad. Nauk.

[47] Grossman JN, Zipfel J (2001). The Meteoritical Bulletin, No. 85, 2001 September. Meteorit. Planet. Sci..

[48] Qian G, Li Y, Gerson AR (2015). Applications of surface analytical techniques in Earth Sciences. Surf. Sci. Rep..

[49] Dera P (2009). Structure and behavior of the barringerite Ni end-member, Ni_2_P, at deep Earth conditions and implications for natural Fe-Ni phosphides in planetary cores. J. Geophys. Res..

[50] Minin, D. A., Shatskiy, A. & Litasov, K. D. Fe–Ni–P phase diagram at 6 GPa. Abstracts of 81st Annual Meeting of the Meteoritical Society. Abstract at, https://www.hou.usra.edu/meetings/metsoc2018/pdf/6174.pdf (2018).

[51] Nisar J, Ahuja R (2010). Structure behavior and equation of state (EOS) of Ni_2_P and (Fe_1-x_Ni_x_)_2_P (allabogdanite) from first principles calculations. Earth Planet. Sci. Lett..

[52] Brandstatter F, Koeberl C, Kurat G (1991). The discovery of iron barringerite in lunar meteorite Y-793274. Geochim. Cosmochim. Acta.

[53] Gounelle M, Zolensky ME, Liou J-C, Bland PA, Alard O (2003). Mineralogy of carbonaceous chondritic microclasts in howardites: identification of C2 fossil micrometeorites. Geochim. Cosmochim. Acta.

[54] Nazarov MA (2009). Phosphorus-bearing sulfides and their associations in CM chondrites. Petrology.

[55] Zucchini A (2018). Chemical and mineralogical characterization of the Mineo (Sicily, Italy) pallasite: A unique sample. Meteorit. Planet. Sci..

[56] Rohrbach A (2007). Metal saturation in the upper mantle. Nature.

[57] Nichols, C. I. O. *et al*. Microstructural and paleomagnetic insight into the cooling history of the IAB parent body. *Geochim. Cosmochim. Acta* **229**, 1–19 (2018).

[58] Bruker APEX2 and SAINT. Bruker AXS Inc., Madison, Wisconsin, USA (2007).

[59] Oxford Diffraction CrysAlis PRO. Oxford Diffraction Ltd, Abingdon, Oxfordshire, England (2006).

[60] Scheldrick GM (2015). Crystal structure refinement with *SHELX*L. Acta Cryst..

[61] Dolomanov OV, Bourhis LJ, Gildea RJ, Howard JA, Puschmann H (2009). OLEX2: a complete structure solution, refinement and analysis program. J. Appl. Cryst..

[62] Carlsson B, Goelin M, Rundqvist S (1973). Determination of the homogenity range and refinement of the crystal structure of Fe_2_P. J. Solid State Chem..

[63] Straumanis ME, Kim DC (1969). Lattice constants, thermal expansion coefficients, densities and perfection of structure of pure iron and iron loaded with hydrogen. Z. Metallkunde.

