# Supplementary material for: Allabogdanite, the high-pressure polymorph of (Fe,Ni)2P, a stishovite-grade indicator of impact processes in the Fe–Ni–P system
Source: Sci Rep. 2019 Jan 31;9:1047. doi: 10.1038/s41598-018-37795-x (PMC6355872; doi:10.1038/s41598-018-37795-x)
Supplement: Supplementary file 1 — Allabogdanite_CIF [file 41598_2018_37795_MOESM1_ESM.pdf]

```
data_Allabogdanite_from_Santa_Catharina_meteorite

_audit_creation_date              2018-08-18
_audit_creation_method
;
Olex2 1.2
(compiled 2017.03.28 svn.r3405 for OlexSys, GUI svn.r5337)
;
_shelx_SHELXL_version_number      '2014/7'


_publ_section_references
;
Dolomanov, O.V., Bourhis, L.J., Gildea, R.J, Howard, J.A.K. & Puschmann, H.
 (2009), J. Appl. Cryst. 42, 339-341.

Sheldrick, G.M. (2015). Acta Cryst. C71, 3-8.
;
_chemical_name_mineral            'Allabogdanite'
_chemical_formula_moiety          '(Fe1.33 Ni0.67 P)'
_chemical_formula_sum             'Fe1.33 Ni0.67 P'
_chemical_formula_weight          144.58


loop_
  _atom_type_symbol
  _atom_type_description
  _atom_type_scat_dispersion_real
  _atom_type_scat_dispersion_imag
  _atom_type_scat_source
 'Fe' 'Fe' 0.3463 0.8444 'International Tables Vol C Tables 4.2.6.8 and 6.1.1.4'
 'Ni' 'Ni' 0.3393 1.1124 'International Tables Vol C Tables 4.2.6.8 and 6.1.1.4'
 'P' 'P' 0.1023 0.0942 'International Tables Vol C Tables 4.2.6.8 and 6.1.1.4'

_shelx_space_group_comment
;
The symmetry employed for this shelxl refinement is uniquely defined
by the following loop, which should always be used as a source of
symmetry information in preference to the above space-group names.
They are only intended as comments.
;
_space_group_crystal_system       'orthorhombic'
_space_group_IT_number            62
_space_group_name_H-M_alt         'P n m a'
_space_group_name_Hall            '-P 2ac 2n'
loop_
  _space_group_symop_operation_xyz
 'x, y, z'
 '-x+1/2, -y, z+1/2'
 '-x, y+1/2, -z'
 'x+1/2, -y+1/2, -z+1/2'
 '-x, -y, -z'
 'x-1/2, y, -z-1/2'
 'x, -y-1/2, z'
 '-x-1/2, y-1/2, z-1/2'

_cell_length_a                    5.7332(7)
_cell_length_b                    3.5413(6)
_cell_length_c                    6.6682(10)
_cell_angle_alpha                 90
_cell_angle_beta                  90
_cell_angle_gamma                 90
_cell_volume                      135.38(3)
_cell_formula_units_Z             4
```

```
_cell_measurement_reflns_used     623
_cell_measurement_temperature     293(2)
_cell_measurement_theta_max       35.49
_cell_measurement_theta_min       4.69
_exptl_absorpt_coefficient_mu     23.964
_exptl_absorpt_correction_T_min   0.00128
_exptl_absorpt_correction_T_max   0.01578
_exptl_absorpt_correction_type    'multi-scan'
_exptl_absorpt_process_details
;
CrysAlisPro 1.171.39.44a (Rigaku Oxford Diffraction, 2018)
Spherical absorption correction
 using equivalent radius and absorption coefficient.
Empirical absorption correction using spherical harmonics,
 implemented in SCALE3 ABSPACK scaling algorithm.
;
_exptl_absorpt_special_details    ?
_exptl_crystal_colour             'metallic white'
_exptl_crystal_density_diffrn     7.093
_exptl_crystal_description        'lamella'
_exptl_crystal_F_000              273
_exptl_crystal_size_max           0.02
_exptl_crystal_size_mid           0.01
_exptl_crystal_size_min           0.005
_diffrn_reflns_av_R_equivalents   0.0164
_diffrn_reflns_av_unetI/netI      0.0076
_diffrn_reflns_Laue_measured_fraction_full 0.979
_diffrn_reflns_Laue_measured_fraction_max 0.981
_diffrn_reflns_limit_h_max        7
_diffrn_reflns_limit_h_min        -7
_diffrn_reflns_limit_k_max        4
_diffrn_reflns_limit_k_min        -4
_diffrn_reflns_limit_l_max        8
_diffrn_reflns_limit_l_min        -8
_diffrn_reflns_number             1075
_diffrn_reflns_point_group_measured_fraction_full 0.979
_diffrn_reflns_point_group_measured_fraction_max 0.981
_diffrn_reflns_theta_full         25.242
_diffrn_reflns_theta_max          25.922
_diffrn_reflns_theta_min          6.524
_diffrn_ambient_temperature       293(2)
_diffrn_measured_fraction_theta_full 0.979
_diffrn_measured_fraction_theta_max 0.981
_diffrn_measurement_device_type   'CCD diffractometer'
_diffrn_radiation_type            MoK\a
_diffrn_radiation_wavelength      0.71073
_diffrn_measurement_method        '\w and \f scans'
_diffrn_source                    ?
_reflns_Friedel_coverage          0.000
_reflns_Friedel_fraction_full     .
_reflns_Friedel_fraction_max      .
_reflns_number_gt                 136
_reflns_number_total              152
_reflns_special_details
;
 Reflections were merged by SHELXL according to the crystal
 class for the calculation of statistics and refinement.

 _reflns_Friedel_fraction is defined as the number of unique
 Friedel pairs measured divided by the number that would be
 possible theoretically, ignoring centric projections and
 systematic absences.
;
_reflns_threshold_expression      'I > 2\s(I)'
```

```
_computing_cell_refinement        'Bruker SAINT'
_computing_data_collection        'Bruker APEX2'
_computing_data_reduction         'CrysAlisPro 1.171.39.44a (Rigaku OD, 2018)'
_computing_molecular_graphics     'Olex2 (Dolomanov et al., 2009)'
_computing_publication_material   'Olex2 (Dolomanov et al., 2009)'
_computing_structure_refinement   'ShelXL (Sheldrick, 2015)'
_computing_structure_solution     'ShelXT (Sheldrick, 2015)'
_refine_diff_density_max          0.946
_refine_diff_density_min          -1.039
_refine_diff_density_rms          0.407
_refine_ls_extinction_coef        .
_refine_ls_extinction_method      none
_refine_ls_goodness_of_fit_ref    1.206
_refine_ls_hydrogen_treatment     undef
_refine_ls_matrix_type            full
_refine_ls_number_parameters      19
_refine_ls_number_reflns          152
_refine_ls_number_restraints      0
_refine_ls_R_factor_all           0.0455
_refine_ls_R_factor_gt            0.0426
_refine_ls_restrained_S_all       1.206
_refine_ls_shift/su_max           0.000
_refine_ls_shift/su_mean          0.000
_refine_ls_structure_factor_coef  Fsqd
_refine_ls_weighting_details
'w=1/[\s^2^(Fo^2^)+(0.0798P)^2^] where P=(Fo^2^+2Fc^2^)/3'
_refine_ls_weighting_scheme       calc
_refine_ls_wR_factor_gt           0.1051
_refine_ls_wR_factor_ref          0.1067
_refine_special_details           ?
_olex2_refinement_description
;
1. Shared sites
{Fe1, Ni1}
{Fe2, Ni2}
2. Uiso/Uaniso restraints and constraints
Uanis(Fe1) = Uanis(Ni1)
Uanis(Fe2) = Uanis(Ni2)
3. Others
 Fixed Sof: Fe1(0.33334) Ni1(0.16667) Fe2(0.33334) Ni2(0.16667)
;
_atom_sites_solution_hydrogens    .
_atom_sites_solution_primary      ?
_atom_sites_solution_secondary    ?
loop_
  _atom_site_label
  _atom_site_type_symbol
  _atom_site_fract_x
  _atom_site_fract_y
  _atom_site_fract_z
  _atom_site_U_iso_or_equiv
  _atom_site_adp_type
  _atom_site_occupancy
  _atom_site_site_symmetry_order
  _atom_site_calc_flag
  _atom_site_refinement_flags_posn
  _atom_site_refinement_flags_adp
  _atom_site_refinement_flags_occupancy
  _atom_site_disorder_assembly
  _atom_site_disorder_group
 Fe1 Fe 0.3583(2) 0.2500 0.9361(2) 0.0279(6) Uani 0.6667 2 d S T P . .
 Ni1 Ni 0.3583(2) 0.2500 0.9361(2) 0.0279(6) Uani 0.3333 2 d S T P . .
 Fe2 Fe 0.4692(2) 0.2500 0.3329(2) 0.0334(7) Uani 0.6667 2 d S T P . .
 Ni2 Ni 0.4692(2) 0.2500 0.3329(2) 0.0334(7) Uani 0.3333 2 d S T P . .
```

```
 P1 P 0.2453(4) 0.2500 0.6227(5) 0.0302(8) Uani 1 2 d S T P . .

loop_
  _atom_site_aniso_label
  _atom_site_aniso_U_11
  _atom_site_aniso_U_22
  _atom_site_aniso_U_33
  _atom_site_aniso_U_23
  _atom_site_aniso_U_13
  _atom_site_aniso_U_12
 Fe1 0.0293(8) 0.0269(9) 0.0273(9) 0.000 -0.0002(5) 0.000
 Ni1 0.0293(8) 0.0269(9) 0.0273(9) 0.000 -0.0002(5) 0.000
 Fe2 0.0360(10) 0.0319(10) 0.0323(10) 0.000 -0.0003(5) 0.000
 Ni2 0.0360(10) 0.0319(10) 0.0323(10) 0.000 -0.0003(5) 0.000
 P1 0.0319(12) 0.0283(14) 0.0306(15) 0.000 0.0011(10) 0.000

_geom_special_details
;
 All esds (except the esd in the dihedral angle between two l.s. planes)
 are estimated using the full covariance matrix.  The cell esds are taken
 into account individually in the estimation of esds in distances, angles
 and torsion angles; correlations between esds in cell parameters are only
 used when they are defined by crystal symmetry.  An approximate (isotropic)
 treatment of cell esds is used for estimating esds involving l.s. planes.
;
loop_
  _geom_bond_atom_site_label_1
  _geom_bond_atom_site_label_2
  _geom_bond_distance
  _geom_bond_site_symmetry_2
  _geom_bond_publ_flag
 Fe1 Fe1 2.5497(18) 5_667 ?
 Fe1 Fe1 2.5497(18) 5_657 ?
 Fe1 Ni1 2.5497(18) 5_657 ?
 Fe1 Ni1 2.5497(18) 5_667 ?
 Fe1 Fe2 2.6708(15) 2_565 ?
 Fe1 Fe2 2.6708(15) 2 ?
 Fe1 Ni2 2.6708(15) 2_565 ?
 Fe1 Ni2 2.6708(15) 2 ?
 Fe1 P1 2.188(3) . ?
 Fe1 P1 2.253(3) 6_657 ?
 Fe1 P1 2.244(2) 2_565 ?
 Fe1 P1 2.244(2) 2 ?
 Fe2 Fe1 2.7079(16) 5_656 ?
 Fe2 Fe1 2.7079(16) 5_666 ?
 Fe2 Fe1 2.6708(15) 2_554 ?
 Fe2 Fe1 2.6708(15) 2_564 ?
 Fe2 Ni1 2.6708(15) 2_554 ?
 Fe2 Ni1 2.7079(16) 5_656 ?
 Fe2 Ni1 2.6708(15) 2_564 ?
 Fe2 P1 2.572(2) 2_554 ?
 Fe2 P1 2.572(2) 2_564 ?
 Fe2 P1 2.319(3) . ?
 Fe2 P1 2.4294(18) 5_666 ?
 Fe2 P1 2.4294(18) 5_656 ?
 P1 Fe1 2.244(2) 2_554 ?
 P1 Fe1 2.253(3) 6_557 ?
 P1 Fe1 2.244(2) 2_564 ?
 P1 Ni1 2.253(3) 6_557 ?
 P1 Ni1 2.244(2) 2_564 ?
 P1 Ni1 2.244(2) 2_554 ?
 P1 Fe2 2.4294(18) 5_666 ?
 P1 Ni2 2.4294(18) 5_666 ?
```

```
loop_
  _geom_angle_atom_site_label_1
  _geom_angle_atom_site_label_2
  _geom_angle_atom_site_label_3
  _geom_angle
  _geom_angle_site_symmetry_1
  _geom_angle_site_symmetry_3
  _geom_angle_publ_flag
 Fe1 Fe1 Fe1 87.97(8) 5_667 5_657 ?
 Fe1 Fe1 Ni1 87.97(8) 5_667 5_657 ?
 Fe1 Fe1 Ni1 0.00(4) 5_667 5_667 ?
 Fe1 Fe1 Fe2 173.94(9) 5_667 2 ?
 Fe1 Fe1 Fe2 94.22(3) 5_657 2 ?
 Fe1 Fe1 Fe2 94.22(3) 5_667 2_565 ?
 Fe1 Fe1 Fe2 173.94(9) 5_657 2_565 ?
 Fe1 Fe1 Ni2 94.22(3) 5_667 2_565 ?
 Fe1 Fe1 Ni2 173.94(9) 5_667 2 ?
 Fe1 Fe1 Ni2 94.22(3) 5_657 2 ?
 Fe1 Fe1 Ni2 173.94(9) 5_657 2_565 ?
 Ni1 Fe1 Fe1 88.0 5_667 5_657 ?
 Ni1 Fe1 Fe1 0.0 5_657 5_657 ?
 Ni1 Fe1 Ni1 87.97(8) 5_667 5_657 ?
 Ni1 Fe1 Fe2 94.22(3) 5_667 2_565 ?
 Ni1 Fe1 Fe2 173.94(9) 5_657 2_565 ?
 Ni1 Fe1 Fe2 94.22(3) 5_657 2 ?
 Ni1 Fe1 Fe2 173.94(9) 5_667 2 ?
 Ni1 Fe1 Ni2 94.22(3) 5_667 2_565 ?
 Ni1 Fe1 Ni2 173.94(9) 5_667 2 ?
 Ni1 Fe1 Ni2 173.94(9) 5_657 2_565 ?
 Ni1 Fe1 Ni2 94.22(3) 5_657 2 ?
 Fe2 Fe1 Fe2 83.05(6) 2 2_565 ?
 Fe2 Fe1 Ni2 83.05(6) 2 2_565 ?
 Ni2 Fe1 Fe2 0.0 2 2 ?
 Ni2 Fe1 Fe2 0.0 2_565 2_565 ?
 Ni2 Fe1 Fe2 83.1 2 2_565 ?
 Ni2 Fe1 Ni2 83.05(6) 2 2_565 ?
 P1 Fe1 Fe1 120.51(8) . 5_657 ?
 P1 Fe1 Fe1 122.11(12) 2_565 5_657 ?
 P1 Fe1 Fe1 55.29(6) 6_657 5_657 ?
 P1 Fe1 Fe1 55.29(6) 6_657 5_667 ?
 P1 Fe1 Fe1 55.64(7) 2 5_657 ?
 P1 Fe1 Fe1 120.51(8) . 5_667 ?
 P1 Fe1 Fe1 122.11(12) 2 5_667 ?
 P1 Fe1 Fe1 55.64(7) 2_565 5_667 ?
 P1 Fe1 Ni1 55.29(6) 6_657 5_667 ?
 P1 Fe1 Ni1 55.64(7) 2 5_657 ?
 P1 Fe1 Ni1 120.51(8) . 5_667 ?
 P1 Fe1 Ni1 120.51(8) . 5_657 ?
 P1 Fe1 Ni1 55.29(6) 6_657 5_657 ?
 P1 Fe1 Ni1 122.11(12) 2_565 5_657 ?
 P1 Fe1 Ni1 122.11(12) 2 5_667 ?
 P1 Fe1 Ni1 55.64(7) 2_565 5_667 ?
 P1 Fe1 Fe2 55.50(7) 2 2 ?
 P1 Fe1 Fe2 130.35(6) 6_657 2_565 ?
 P1 Fe1 Fe2 55.50(7) 2_565 2_565 ?
 P1 Fe1 Fe2 62.99(5) . 2 ?
 P1 Fe1 Fe2 118.67(8) 2_565 2 ?
 P1 Fe1 Fe2 62.99(5) . 2_565 ?
 P1 Fe1 Fe2 130.35(6) 6_657 2 ?
 P1 Fe1 Fe2 118.67(8) 2 2_565 ?
 P1 Fe1 Ni2 118.67(8) 2_565 2 ?
 P1 Fe1 Ni2 55.50(7) 2_565 2_565 ?
 P1 Fe1 Ni2 130.35(6) 6_657 2_565 ?
 P1 Fe1 Ni2 55.50(7) 2 2 ?
```

P1 Fe1 Ni2 118.67(8) 2 2_565 ?
P1 Fe1 Ni2 130.35(6) 6_657 2 ?
P1 Fe1 Ni2 62.99(5) . 2 ?
P1 Fe1 Ni2 62.99(5) . 2_565 ?
P1 Fe1 P1 97.19(9) . 6_657 ?
P1 Fe1 P1 116.82(6) . 2_565 ?
P1 Fe1 P1 110.93(8) 2 6_657 ?
P1 Fe1 P1 104.19(14) 2_565 2 ?
P1 Fe1 P1 116.82(6) . 2 ?
P1 Fe1 P1 110.93(8) 2_565 6_657 ?
Fe1 Fe2 Fe1 83.05(6) 2_554 2_564 ?
Fe1 Fe2 Fe1 149.42(7) 2_554 5_666 ?
Fe1 Fe2 Fe1 89.65(4) 2_554 5_656 ?
Fe1 Fe2 Fe1 81.67(6) 5_656 5_666 ?
Fe1 Fe2 Fe1 89.65(4) 2_564 5_666 ?
Fe1 Fe2 Fe1 149.42(7) 2_564 5_656 ?
Fe1 Fe2 Ni1 89.65(4) 2_554 5_656 ?
Fe1 Fe2 Ni1 0.00(7) 2_554 2_554 ?
Fe1 Fe2 Ni1 0.00(7) 2_564 2_564 ?
Fe1 Fe2 Ni1 149.42(7) 2_564 5_656 ?
Fe1 Fe2 Ni1 0.00(7) 5_656 5_656 ?
Fe1 Fe2 Ni1 83.05(6) 2_554 2_564 ?
Ni1 Fe2 Fe1 89.7 2_554 5_656 ?
Ni1 Fe2 Fe1 149.4 2_554 5_666 ?
Ni1 Fe2 Fe1 83.1 2_554 2_564 ?
Ni1 Fe2 Fe1 81.7 5_656 5_666 ?
Ni1 Fe2 Fe1 89.7 2_564 5_666 ?
Ni1 Fe2 Fe1 149.4 2_564 5_656 ?
Ni1 Fe2 Ni1 83.05(6) 2_554 2_564 ?
Ni1 Fe2 Ni1 149.42(7) 2_564 5_656 ?
Ni1 Fe2 Ni1 89.65(4) 2_554 5_656 ?
P1 Fe2 Fe1 157.73(11) 5_666 2_554 ?
P1 Fe2 Fe1 105.29(8) 2_564 5_656 ?
P1 Fe2 Fe1 50.45(6) 2_564 5_666 ?
P1 Fe2 Fe1 87.65(5) 5_656 2_554 ?
P1 Fe2 Fe1 108.13(8) 5_656 5_666 ?
P1 Fe2 Fe1 49.30(7) 2_554 2_554 ?
P1 Fe2 Fe1 50.45(6) 2_554 5_656 ?
P1 Fe2 Fe1 52.88(5) . 2_564 ?
P1 Fe2 Fe1 50.06(8) 5_666 5_666 ?
P1 Fe2 Fe1 108.13(8) 5_666 5_656 ?
P1 Fe2 Fe1 105.29(8) 2_554 5_666 ?
P1 Fe2 Fe1 157.73(11) 5_656 2_564 ?
P1 Fe2 Fe1 49.30(7) 2_564 2_564 ?
P1 Fe2 Fe1 50.06(8) 5_656 5_656 ?
P1 Fe2 Fe1 52.88(5) . 2_554 ?
P1 Fe2 Fe1 105.11(7) 2_554 2_564 ?
P1 Fe2 Fe1 105.11(7) 2_564 2_554 ?
P1 Fe2 Fe1 138.94(3) . 5_666 ?
P1 Fe2 Fe1 138.94(3) . 5_656 ?
P1 Fe2 Fe1 87.65(5) 5_666 2_564 ?
P1 Fe2 Ni1 138.94(3) . 5_656 ?
P1 Fe2 Ni1 105.11(7) 2_554 2_564 ?
P1 Fe2 Ni1 49.30(7) 2_554 2_554 ?
P1 Fe2 Ni1 108.13(8) 5_666 5_656 ?
P1 Fe2 Ni1 52.88(5) . 2_554 ?
P1 Fe2 Ni1 50.06(8) 5_656 5_656 ?
P1 Fe2 Ni1 157.73(11) 5_656 2_564 ?
P1 Fe2 Ni1 50.45(6) 2_554 5_656 ?
P1 Fe2 Ni1 49.30(7) 2_564 2_564 ?
P1 Fe2 Ni1 105.29(8) 2_564 5_656 ?
P1 Fe2 Ni1 87.65(5) 5_656 2_554 ?
P1 Fe2 Ni1 52.88(5) . 2_564 ?
P1 Fe2 Ni1 105.11(7) 2_564 2_554 ?

```
 P1 Fe2 Ni1 87.65(5) 5_666 2_564 ?
 P1 Fe2 Ni1 157.73(11) 5_666 2_554 ?
 P1 Fe2 P1 152.93(8) 5_656 2_564 ?
 P1 Fe2 P1 83.50(6) 5_656 2_554 ?
 P1 Fe2 P1 105.74(9) . 5_656 ?
 P1 Fe2 P1 87.02(9) 2_554 2_564 ?
 P1 Fe2 P1 105.74(9) . 5_666 ?
 P1 Fe2 P1 93.58(9) 5_666 5_656 ?
 P1 Fe2 P1 100.93(5) . 2_564 ?
 P1 Fe2 P1 100.93(5) . 2_554 ?
 P1 Fe2 P1 152.93(8) 5_666 2_554 ?
 P1 Fe2 P1 83.50(6) 5_666 2_564 ?
 Fe1 P1 Fe1 104.19(14) 2_554 2_564 ?
 Fe1 P1 Fe1 69.07(8) 2_564 6_557 ?
 Fe1 P1 Fe1 69.07(8) 2_554 6_557 ?
 Fe1 P1 Fe1 127.44(7) . 2_554 ?
 Fe1 P1 Fe1 117.23(13) . 6_557 ?
 Fe1 P1 Fe1 127.44(7) . 2_564 ?
 Fe1 P1 Ni1 117.2 . 6_557 ?
 Fe1 P1 Ni1 104.19(14) 2_554 2_564 ?
 Fe1 P1 Ni1 127.4 . 2_564 ?
 Fe1 P1 Ni1 69.07(8) 2_554 6_557 ?
 Fe1 P1 Ni1 69.07(8) 2_564 6_557 ?
 Fe1 P1 Ni1 127.4 . 2_554 ?
 Fe1 P1 Fe2 145.19(14) 2_554 5_666 ?
 Fe1 P1 Fe2 113.57(14) 6_557 . ?
 Fe1 P1 Fe2 70.78(5) 2_564 5_666 ?
 Fe1 P1 Fe2 129.19(13) . . ?
 Fe1 P1 Fe2 71.59(8) . 5_666 ?
 Fe1 P1 Fe2 71.62(9) 2_554 . ?
 Fe1 P1 Fe2 71.62(9) 2_564 . ?
 Fe1 P1 Fe2 133.21(5) 6_557 5_666 ?
 Fe1 P1 Ni2 71.59(8) . 5_666 ?
 Fe1 P1 Ni2 133.21(5) 6_557 5_666 ?
 Fe1 P1 Ni2 145.19(14) 2_554 5_666 ?
 Fe1 P1 Ni2 70.78(5) 2_564 5_666 ?
 Ni1 P1 Fe1 0.0 6_557 6_557 ?
 Ni1 P1 Fe1 69.1 2_564 6_557 ?
 Ni1 P1 Fe1 69.1 2_554 6_557 ?
 Ni1 P1 Fe1 104.2 2_554 2_564 ?
 Ni1 P1 Fe1 0.0 2_554 2_554 ?
 Ni1 P1 Fe1 0.0 2_564 2_564 ?
 Ni1 P1 Ni1 104.19(14) 2_554 2_564 ?
 Ni1 P1 Ni1 69.07(8) 2_564 6_557 ?
 Ni1 P1 Ni1 69.07(8) 2_554 6_557 ?
 Ni1 P1 Fe2 71.62(9) 2_554 . ?
 Ni1 P1 Fe2 71.62(9) 2_564 . ?
 Ni1 P1 Fe2 133.21(5) 6_557 5_666 ?
 Ni1 P1 Fe2 113.57(14) 6_557 . ?
 Ni1 P1 Fe2 70.78(5) 2_564 5_666 ?
 Ni1 P1 Fe2 145.19(14) 2_554 5_666 ?
 Ni1 P1 Ni2 70.78(5) 2_564 5_666 ?
 Ni1 P1 Ni2 145.19(14) 2_554 5_666 ?
 Ni1 P1 Ni2 133.21(5) 6_557 5_666 ?
 Fe2 P1 Fe2 74.26(9) . 5_666 ?
 Fe2 P1 Ni2 74.3 . 5_666 ?
 Ni2 P1 Fe2 0.0 5_666 5_666 ?

_shelx_res_file
;
   allabogdanite_sc.res created by SHELXL-2014/7

TITL Allabogdanite_SC in Pnma
CELL 0.71073 5.7332 3.5413 6.6682 90 90 90
```

```
ZERR 4 0.0007 0.0006 0.001 0 0 0
LATT 1
SYMM 0.5-X,-Y,0.5+Z
SYMM -X,0.5+Y,-Z
SYMM 0.5+X,0.5-Y,0.5-Z
SFAC Fe Ni P
UNIT 5.33 2.67 4
EADP Fe1 Ni1
EADP Fe2 Ni2
EXYZ Fe1 Ni1
EXYZ Fe2 Ni2

L.S. 20
PLAN  10
BOND
LIST 6
fmap 2
acta
OMIT -4 52
OMIT 1 0 1
OMIT 0 0 2
OMIT 1 0 2
REM <olex2.extras>
REM <HklSrc "%.\\Allabogdanite_SC.hkl">
REM </olex2.extras>

WGHT    0.079800
FVAR       0.65521
FE1   1    0.358302    0.250000    0.936126    10.33334    0.02935    0.02690 =
         0.02731    0.00000   -0.00025    0.00000
NI1   2    0.358302    0.250000    0.936126    10.16667    0.02935    0.02690 =
         0.02731    0.00000   -0.00025    0.00000
FE2   1    0.469166    0.250000    0.332945    10.33334    0.03598    0.03189 =
         0.03227    0.00000   -0.00026    0.00000
NI2   2    0.469166    0.250000    0.332945    10.16667    0.03598    0.03189 =
         0.03227    0.00000   -0.00026    0.00000
P1    3    0.245348    0.250000    0.622670    10.50000    0.03186    0.02825 =
         0.03058    0.00000    0.00105    0.00000
HKLF 4

REM  Allabogdanite_SC in Pnma
REM R1 =  0.0426 for     136 Fo > 4sig(Fo)  and  0.0455 for all     152 data
REM     19 parameters refined using      0 restraints

END

WGHT      0.0798      0.0000

REM Highest difference peak  0.946,  deepest hole -1.039,  1-sigma level  0.407
Q1    1   0.2645  0.2500  0.9072  10.50000  0.05    0.95
Q2    1   0.4296  0.1212  0.9063  11.00000  0.05    0.92
Q3    1   0.5131  0.2500  0.9282  10.50000  0.05    0.92
Q4    1   0.6244  0.2500  0.3162  10.50000  0.05    0.92
Q5    1   0.3255  0.2500  1.0345  10.50000  0.05    0.79
Q6    1   0.5306  0.0500  0.2950  11.00000  0.05    0.77
Q7    1   0.5301  0.1256  0.4194  11.00000  0.05    0.65
Q8    1   0.3954  0.0590  0.3357  11.00000  0.05    0.64
Q9    1   0.3238  0.2500  0.2850  10.50000  0.05    0.60
Q10   1   0.3852  0.2500  0.6526  10.50000  0.05    0.47
;
_shelx_res_checksum              160
_shelx_hkl_file
;
   0   0   2  293.06    7.70
```

0	0	-2	300.00	10.95
0	0	-3	1.86	6.40
0	0	-3	-2.49	7.60
0	0	3	3.37	4.92
0	0	-4	1452.64	14.12
0	0	-4	1487.41	16.93
0	0	4	1471.76	12.61
0	0	5	1.50	3.55
0	0	-5	7.48	5.93
0	0	-5	1.02	4.53
0	0	-6	6.59	5.35
0	0	6	7.49	3.36
0	0	-6	12.07	4.30
0	0	-7	9.82	4.92
0	0	-7	-2.87	3.78
0	0	-8	160.83	6.60
0	0	-9	4.50	4.25
0	0	-10	55.84	4.67
0	0	-11	1.09	3.49
1	0	0	-3.20	11.01
1	0	0	-3.21	8.17
-1	0	0	-3.78	7.59
-1	0	1	114.15	9.62
1	0	-1	109.27	11.53
1	0	1	119.67	6.21
1	0	1	112.73	9.37
1	0	-1	93.85	11.97
-1	0	-1	114.40	7.89
1	0	2	256.50	6.16
-1	0	2	253.62	9.58
-1	0	-2	247.23	8.85
1	0	-2	251.95	11.36
1	0	-2	255.29	12.65
-1	0	-3	3347.48	21.84
-1	0	3	3338.35	21.98
1	0	-3	3332.62	23.55
1	0	3	3335.29	15.59
1	0	-3	3335.05	26.75
-1	0	4	486.24	9.19
1	0	4	481.55	6.48
-1	0	-4	479.61	9.78
1	0	-4	498.20	10.37
1	0	-4	471.75	11.88
1	0	-5	454.52	10.98
-1	0	-5	436.74	9.22
1	0	5	469.53	6.19
-1	0	5	472.54	8.27
1	0	-5	461.25	9.34
-1	0	6	503.27	7.89
-1	0	-6	490.78	8.13
1	0	-6	482.37	10.72
1	0	-6	499.00	9.04
-1	0	-6	499.15	9.57
-1	0	7	276.06	5.92
-1	0	-7	279.02	6.38
1	0	-7	299.44	8.59
1	0	-7	282.16	6.99
-1	0	-7	281.86	7.71
1	0	-8	0.32	4.70
-1	0	-8	9.13	4.27
-1	0	-9	21.39	4.32
1	0	-9	17.16	4.75
1	0	-10	86.39	5.42
-1	0	-10	98.24	5.12

-1	0	-11	17.35	3.66
1	0	-11	20.79	3.92
2	0	0	151.32	9.90
2	0	0	125.67	13.85
-2	0	0	126.32	9.66
2	0	0	133.93	8.37
2	0	1	11.93	8.29
2	0	1	12.10	6.09
-2	0	1	10.17	8.89
2	0	-1	13.09	12.27
-2	0	-1	9.99	7.29
2	0	-1	18.92	10.33
2	0	-1	20.35	6.99
-2	0	2	958.92	15.32
-2	0	-2	955.14	13.04
2	0	2	967.89	9.53
2	0	-2	953.36	17.44
2	0	-2	945.17	17.24
-2	0	-3	1313.11	13.66
2	0	-3	1324.25	18.99
2	0	3	1344.55	9.60
-2	0	3	1332.79	16.24
2	0	-3	1352.36	17.46
2	0	-4	12.18	7.61
-2	0	-4	7.89	4.89
-2	0	4	2.64	5.34
2	0	-4	12.22	6.41
2	0	4	3.93	2.56
-2	0	-5	907.91	11.40
2	0	5	908.93	6.97
2	0	-5	900.57	14.77
2	0	-5	924.73	13.18
-2	0	5	914.79	12.03
-2	0	6	409.81	8.19
2	0	-6	393.52	10.35
-2	0	-6	419.81	8.30
2	0	-6	415.54	9.12
2	0	-7	2.19	3.96
-2	0	7	0.31	3.52
-2	0	-7	2.03	3.98
2	0	-7	2.52	5.23
2	0	-8	83.50	6.15
-2	0	-8	82.53	4.85
-2	0	-9	14.57	3.71
2	0	-9	10.34	4.48
-2	0	-10	4.92	3.26
2	0	-10	3.19	4.12
2	0	-11	19.16	4.03
-2	0	-11	12.86	3.46
-3	0	0	0.76	6.76
3	0	0	-2.17	5.99
3	0	0	1.85	6.80
3	0	0	1.22	9.99
3	0	-1	2790.32	18.82
3	0	1	2811.51	21.20
3	0	1	2833.40	18.61
-3	0	-1	2800.34	21.16
3	0	1	2795.82	27.41
3	0	-1	2822.74	24.71
3	0	-1	2755.35	28.11
-3	0	1	2793.01	24.02
3	0	-2	2185.37	23.14
3	0	2	2203.26	20.93
3	0	2	2225.03	14.22

3	0	-2	2207.74	24.59
-3	0	2	2232.04	21.29
-3	0	-2	2224.39	18.65
3	0	-2	2220.49	16.22
3	0	-3	238.97	10.42
3	0	-3	242.04	10.37
-3	0	-3	238.45	7.14
3	0	3	249.09	4.92
-3	0	3	218.62	8.81
-3	0	-4	3.84	4.18
3	0	4	1.90	2.33
3	0	-4	2.75	6.37
-3	0	4	-1.95	5.27
3	0	-4	6.19	7.27
-3	0	5	4.98	4.73
-3	0	-5	-5.41	3.97
3	0	-5	1.21	6.81
3	0	-5	12.63	5.93
-3	0	-6	12.52	3.85
3	0	-6	17.14	6.48
3	0	-6	14.76	5.11
-3	0	6	14.31	4.10
-3	0	7	39.67	3.91
-3	0	-7	35.44	4.09
3	0	-7	30.97	5.85
3	0	-7	31.30	4.76
3	0	-8	14.77	5.16
-3	0	-8	15.16	3.50
3	0	-9	172.69	7.30
-3	0	-9	175.18	5.61
-3	0	-10	5.83	3.24
3	0	-10	2.78	4.16
3	0	-11	0.29	3.72
4	0	0	53.41	9.08
4	0	0	38.63	5.58
4	0	0	52.97	6.19
-4	0	0	50.19	6.34
4	0	-1	441.81	10.95
-4	0	-1	448.10	9.63
4	0	-1	397.82	7.91
4	0	-1	427.28	13.69
4	0	1	438.71	9.62
-4	0	1	432.54	10.44
4	0	1	433.43	12.63
4	0	1	452.31	8.28
4	0	2	752.20	12.36
4	0	-2	733.31	15.25
4	0	2	719.62	8.71
-4	0	-2	711.57	11.15
-4	0	2	710.53	12.54
4	0	-2	729.39	13.40
-4	0	-3	65.89	5.80
-4	0	3	66.45	6.48
4	0	-3	57.21	5.01
4	0	-3	58.92	7.89
4	0	-3	68.72	7.88
-4	0	-3	67.91	5.46
4	0	3	73.33	3.54
-4	0	4	269.76	8.40
4	0	-4	252.30	9.77
-4	0	-4	281.46	6.95
4	0	-4	258.51	10.10
-4	0	5	6.31	4.71
4	0	-5	10.94	6.72

-4	0	-5	4.23	3.68
4	0	-5	6.86	5.96
-4	0	-6	321.57	6.83
4	0	-6	326.63	9.94
-4	0	6	313.95	7.66
4	0	-6	310.01	8.99
4	0	-7	68.52	6.33
4	0	-7	60.67	5.38
-4	0	-7	75.42	4.21
-4	0	7	74.32	4.44
-4	0	-8	108.21	4.58
4	0	-8	116.35	6.80
-4	0	-9	0.53	3.00
4	0	-9	3.17	4.49
4	0	-10	-1.56	4.03
5	0	0	3.41	4.44
-5	0	0	2.03	5.14
5	0	0	5.34	7.75
5	0	0	-0.19	4.32
5	0	1	99.86	6.01
5	0	-1	95.47	6.59
5	0	1	92.99	4.94
5	0	-1	80.23	8.80
5	0	-1	77.19	4.91
-5	0	1	87.46	6.45
-5	0	-1	97.23	6.34
5	0	-2	125.56	9.37
5	0	-2	133.77	7.57
5	0	2	143.76	4.86
-5	0	-2	129.82	6.45
-5	0	2	140.11	6.92
-5	0	3	44.68	5.80
-5	0	-3	43.91	4.87
-5	0	-3	34.32	5.10
5	0	-3	45.96	6.68
5	0	-3	42.93	7.22
5	0	3	51.94	3.21
-5	0	-4	690.98	10.02
5	0	-4	684.36	13.51
-5	0	4	695.70	11.41
-5	0	5	5.08	4.21
-5	0	-5	5.84	3.50
5	0	-5	16.24	5.43
5	0	-5	11.50	5.90
5	0	-6	-7.24	4.84
-5	0	-6	0.29	3.32
-5	0	6	-0.88	3.76
-5	0	7	1.68	3.35
5	0	-7	-1.56	4.27
-5	0	-7	12.72	3.20
5	0	-7	6.10	5.12
-5	0	-8	40.12	3.63
5	0	-8	26.25	5.09
5	0	-9	22.27	4.82
5	0	-10	-3.32	3.99
6	0	0	41.85	4.46
6	0	0	48.01	4.45
6	0	0	52.03	7.61
-6	0	0	44.84	5.30
-6	0	-1	302.60	8.03
6	0	-1	326.10	8.13
6	0	1	320.12	6.83
-6	0	1	304.66	8.16
6	0	-1	315.89	10.78

6	0	1	309.52	7.59
6	0	2	7.20	2.91
-6	0	-2	20.63	4.50
-6	0	2	11.63	4.77
6	0	-2	18.38	5.19
6	0	-2	19.31	6.57
-6	0	-3	117.68	5.55
-6	0	3	117.80	6.22
6	0	-3	113.79	7.81
-6	0	-3	130.66	6.14
6	0	-3	116.96	6.89
-6	0	-4	21.38	4.03
6	0	-4	13.21	5.73
6	0	-4	17.89	5.43
-6	0	4	15.19	4.47
-6	0	-5	-4.11	3.49
6	0	-5	3.51	5.27
6	0	-5	1.79	5.28
-6	0	5	6.44	3.89
6	0	-6	-5.88	5.03
6	0	-6	-1.16	4.65
-6	0	6	2.19	3.55
-6	0	-6	3.60	3.23
-6	0	-7	45.50	3.68
-6	0	7	48.17	3.85
6	0	-7	39.10	5.34
6	0	-7	46.49	5.12
6	0	-8	74.00	5.55
-6	0	-8	85.54	4.13
6	0	-9	-5.73	3.95
7	0	0	1.59	3.31
-7	0	0	-1.00	4.09
7	0	-1	73.67	4.79
7	0	-1	91.66	7.37
-7	0	1	88.55	5.48
-7	0	-1	76.90	5.18
7	0	1	75.13	3.83
-7	0	2	200.27	6.81
-7	0	-2	193.78	6.80
7	0	-2	187.56	8.57
7	0	-2	192.16	6.76
7	0	-3	38.15	4.87
7	0	-3	32.41	6.03
-7	0	-3	44.20	4.40
-7	0	3	36.56	4.60
-7	0	4	61.44	4.86
7	0	-4	48.41	5.96
7	0	-4	59.76	5.22
-7	0	-4	55.02	4.23
-7	0	-5	12.82	3.55
-7	0	5	9.46	3.70
7	0	-5	14.04	4.52
7	0	-5	12.09	4.82
-7	0	6	8.85	3.33
7	0	-6	4.55	4.41
7	0	-6	8.55	4.28
-7	0	-6	15.97	3.58
7	0	-7	52.91	5.12
-7	0	7	51.54	4.03
-7	0	-7	51.83	3.85
7	0	-8	19.39	4.18
-8	0	0	112.28	5.43
8	0	-1	8.75	3.20
-8	0	1	5.49	3.74

-8	0	-1	17.73	3.90
8	0	-2	8.32	5.44
8	0	-2	21.25	3.73
-8	0	-2	14.11	4.03
-8	0	-2	20.12	3.83
-8	0	2	14.86	4.02
-8	0	3	34.34	4.27
-8	0	-3	40.16	3.99
8	0	-3	31.44	5.63
8	0	-3	32.68	4.26
8	0	-4	12.02	3.94
8	0	-4	14.41	5.16
-8	0	-4	19.26	3.59
-8	0	4	16.37	3.83
8	0	-5	84.43	5.94
8	0	-5	105.84	5.40
-8	0	5	91.10	4.98
-8	0	-5	97.60	4.59
-8	0	-6	8.18	3.20
-8	0	6	13.60	3.31
8	0	-6	8.82	4.44
8	0	-6	16.02	4.05
8	0	-7	13.27	3.71
-8	0	-7	4.70	2.88
-8	0	7	11.14	2.90
-9	0	0	3.35	3.35
-9	0	-1	-3.77	3.39
-9	0	-1	0.24	3.08
-9	0	1	-1.17	3.29
-9	0	2	39.52	4.15
-9	0	-2	37.35	3.86
-9	0	3	7.47	3.51
9	0	-3	7.61	3.14
-9	0	4	2.31	3.36
-9	0	-4	4.73	3.02
9	0	-4	3.67	3.07
-9	0	5	-2.14	3.13
-9	0	-5	4.57	3.08
9	0	-5	8.31	3.33
-10	0	0	56.21	4.15
-10	0	0	69.09	4.11
-10	0	1	7.29	3.19
-10	0	-1	9.70	3.00
0	1	0	-1.63	7.44
0	-1	0	-0.73	6.35
0	1	-1	37.32	10.65
0	-1	1	41.76	7.15
0	1	1	33.47	8.99
0	1	2	3.42	7.48
0	-1	2	-0.64	6.18
0	1	-2	4.44	9.66
0	1	-3	3509.33	26.56
0	-1	3	3539.56	22.00
0	1	-3	3471.25	28.32
0	-1	-3	3522.08	17.62
0	-1	-4	1.86	2.66
0	-1	4	0.55	4.51
0	1	-4	10.21	6.73
0	1	-4	1.67	7.55
0	-1	-4	1.36	4.47
0	1	-5	1320.37	17.32
0	-1	5	1342.14	12.93
0	-1	-5	1365.03	13.28
0	1	-5	1389.05	15.57

0	-1	-5	1311.34	8.75
0	1	-6	2.68	4.72
0	-1	6	2.53	3.30
0	-1	-6	1.07	4.46
0	1	-6	0.02	6.17
0	-1	7	195.67	5.10
0	1	-7	214.71	8.15
0	1	-7	194.07	6.76
0	-1	-7	214.17	6.78
0	-1	8	0.30	2.64
0	1	-8	6.85	3.98
0	-1	-8	3.02	3.89
0	1	-8	6.54	5.06
0	1	-9	-0.42	4.40
0	-1	-9	3.16	3.63
0	1	-10	-0.32	3.98
0	1	-11	7.54	3.65
-1	1	0	2.59	8.86
1	-1	0	3.99	8.57
1	1	0	4.00	10.71
-1	-1	0	0.83	4.77
1	1	0	0.21	9.58
-1	1	1	319.82	8.81
1	1	-1	315.13	11.62
-1	1	-1	344.79	12.16
1	-1	1	329.12	10.15
1	1	-1	305.75	12.71
-1	-1	1	323.41	8.42
1	-1	-1	319.14	10.93
1	1	1	329.11	12.02
1	1	1	316.19	9.79
1	1	-2	4729.67	31.88
-1	1	2	4710.06	20.31
1	-1	2	4730.51	25.71
1	1	-2	4702.85	32.19
-1	1	-2	4725.34	31.25
-1	-1	2	4733.41	25.44
1	1	2	4726.99	29.89
1	-1	-2	4745.63	27.57
-1	1	3	1153.26	9.97
1	-1	-3	1123.69	14.41
1	1	-3	1143.90	17.70
1	-1	-3	1124.37	10.88
-1	1	-3	1104.25	16.63
-1	-1	3	1094.32	13.38
-1	-1	-3	1130.23	6.97
1	1	-3	1108.02	16.43
1	-1	3	1111.41	12.72
1	1	3	1117.23	16.32
-1	-1	-4	28.99	3.52
-1	1	-4	23.14	7.12
1	-1	-4	32.01	6.28
1	-1	4	29.48	4.57
1	1	-4	24.87	6.89
-1	1	4	28.13	3.35
1	1	-4	30.29	8.02
-1	-1	4	33.93	5.15
1	-1	-4	28.38	4.51
-1	-1	-5	-0.64	2.50
1	-1	-5	-2.20	3.55
-1	-1	5	4.77	4.20
1	-1	5	2.32	3.52
1	1	-5	5.53	6.09
1	-1	-5	1.85	5.39

1	1	-5	4.27	7.24
-1	1	-5	4.24	6.15
-1	-1	-5	1.62	3.70
-1	1	-6	69.24	6.47
-1	-1	6	64.04	4.55
1	-1	6	71.95	4.04
-1	-1	-6	70.47	4.58
1	-1	-6	56.83	5.63
1	1	-6	75.97	7.48
1	1	-6	71.36	5.99
-1	1	-6	79.11	5.52
-1	1	-7	310.03	7.46
1	1	-7	302.47	8.03
1	-1	-7	313.18	8.16
-1	-1	-7	324.53	7.06
-1	1	-7	344.80	8.91
1	1	-7	338.30	9.71
1	-1	7	323.65	5.73
-1	-1	7	310.26	6.51
1	-1	-8	80.41	5.36
-1	-1	8	74.42	3.88
1	1	-8	84.01	6.49
-1	1	-8	82.30	5.75
-1	-1	-8	72.74	4.52
-1	1	-8	78.45	4.70
1	1	-8	76.26	5.20
-1	1	-9	75.99	5.34
1	1	-9	85.18	6.01
1	-1	-9	89.42	5.33
1	1	-9	72.03	4.66
1	-1	-10	89.27	5.07
1	1	-10	83.05	5.60
-1	1	-10	82.83	5.17
1	1	-11	-5.04	3.69
-1	1	-11	-3.68	3.45
2	1	0	4918.59	21.21
2	-1	0	4914.09	30.01
-2	1	0	4923.00	29.13
2	1	0	4922.38	33.28
2	1	0	4913.94	33.19
-2	-1	0	4921.88	21.24
2	1	-1	3410.62	29.14
2	1	1	3553.12	26.97
2	1	1	3487.38	25.91
-2	-1	1	3479.69	21.00
2	1	-1	3463.47	25.29
-2	-1	-1	3468.97	14.61
2	-1	-1	3442.64	26.50
2	1	-1	3478.10	26.47
2	-1	1	3456.42	24.14
-2	1	-1	3481.71	25.52
-2	1	1	3425.23	24.45
2	1	2	457.63	11.92
-2	-1	2	429.24	9.50
2	1	-2	447.55	12.82
-2	1	-2	487.62	11.77
2	-1	-2	457.93	12.51
2	1	2	473.40	10.13
-2	1	2	491.97	10.29
2	-1	-2	471.92	11.03
-2	-1	-2	471.89	5.32
2	1	-2	450.33	13.20
2	-1	2	488.20	9.59
2	-1	3	377.18	7.90

2	1	-3	361.55	11.64
-2	-1	-3	376.43	5.20
-2	1	3	351.89	8.55
-2	1	-3	380.35	10.65
2	1	-3	374.94	11.75
2	-1	-3	363.14	9.53
2	1	3	378.74	11.08
-2	-1	3	381.52	8.86
2	-1	-3	382.28	11.50
2	1	-4	26.13	7.18
2	-1	-4	23.54	6.81
-2	-1	-4	28.14	2.96
-2	1	4	27.23	4.41
-2	-1	4	24.16	5.03
2	1	-4	32.79	7.90
2	-1	4	34.55	4.09
2	-1	-4	32.88	5.69
-2	1	-4	32.18	6.51
2	-1	5	405.99	6.84
-2	-1	5	407.93	8.67
2	1	-5	386.00	10.33
-2	1	5	403.00	7.26
-2	-1	-5	408.58	6.31
2	-1	-5	410.60	8.34
2	-1	-5	435.02	10.70
-2	1	-5	394.69	9.72
2	1	-5	404.01	11.50
-2	-1	6	45.24	4.75
-2	1	-6	45.13	5.72
-2	-1	-6	41.88	3.64
2	1	-6	41.18	7.06
2	-1	-6	55.32	6.10
2	-1	6	50.82	3.37
2	1	-6	47.78	5.71
-2	-1	7	24.53	3.84
2	-1	-7	35.05	5.18
2	1	-7	23.61	4.96
2	-1	7	33.80	2.87
2	1	-7	32.45	6.29
-2	1	-7	32.63	4.95
2	1	-8	187.38	7.91
-2	1	-8	181.85	6.66
-2	-1	8	174.71	5.26
-2	1	-8	173.39	5.63
2	-1	-8	185.36	7.14
2	1	-8	177.61	6.81
-2	1	-9	5.38	3.94
2	-1	-9	1.79	4.10
2	1	-9	9.25	4.72
2	1	-10	34.39	4.76
-2	1	-10	28.30	3.89
2	-1	-10	27.49	4.26
2	1	-11	0.47	3.81
2	-1	-11	2.83	3.42
-3	1	0	3.82	7.09
3	1	0	1.72	7.50
3	-1	0	-2.72	6.67
-3	-1	0	4.60	4.68
3	1	0	-0.91	4.68
3	1	0	-1.86	8.87
-3	1	-1	354.20	9.95
3	-1	-1	324.17	10.52
3	1	-1	328.81	9.61
3	1	1	331.70	11.30

3	-1	1	331.71	8.98
-3	-1	1	338.16	8.58
3	1	-1	329.80	12.56
-3	1	1	310.69	10.25
3	1	-1	321.55	10.87
3	1	1	336.85	10.67
-3	-1	-1	332.25	6.98
3	1	2	-0.92	7.09
3	1	-2	-1.70	6.95
3	-1	-2	-1.29	7.31
-3	1	-2	2.64	5.94
-3	1	2	-2.13	6.11
-3	-1	2	2.28	5.25
3	-1	-2	-2.53	7.31
3	1	-2	1.31	6.41
3	-1	2	1.55	4.91
3	1	-2	3.38	8.43
-3	-1	-2	-0.70	3.55
3	1	2	-5.20	6.46
3	1	3	107.13	7.95
3	-1	3	91.00	5.04
-3	-1	3	97.10	6.52
-3	-1	-3	98.10	4.15
3	1	-3	83.07	8.49
-3	1	3	86.82	6.63
3	-1	-3	92.16	7.88
3	-1	-3	111.09	8.55
3	1	-3	83.10	8.58
-3	1	-3	99.47	6.90
-3	1	-4	1395.83	15.63
-3	-1	4	1423.97	15.44
3	-1	-4	1410.78	18.10
-3	1	4	1428.14	14.42
3	1	-4	1414.80	18.33
3	-1	-4	1407.14	16.29
3	-1	4	1394.47	12.06
3	1	-4	1400.70	18.17
3	1	4	1411.72	17.52
-3	-1	-4	1414.71	10.58
3	-1	-5	279.56	9.68
3	-1	-5	261.42	7.76
-3	-1	5	274.02	7.84
-3	1	-5	261.01	7.90
-3	1	5	271.97	6.92
-3	-1	-5	274.73	5.37
3	1	-5	279.30	10.01
3	1	-5	272.28	9.25
3	-1	5	281.62	5.61
-3	-1	6	59.60	5.19
3	1	-6	66.66	6.27
-3	1	-6	78.08	5.54
-3	1	6	62.55	4.32
3	1	-6	72.56	7.39
3	-1	-6	82.27	6.65
3	-1	6	82.31	3.52
3	-1	-7	17.63	5.23
-3	-1	7	23.24	4.02
3	1	-7	14.59	5.22
-3	1	-7	20.63	4.55
3	1	-7	9.08	5.64
3	1	-8	48.19	4.81
-3	1	-8	49.77	4.59
3	1	-8	62.40	6.04
3	-1	-8	50.16	5.55

-3	-1	8	50.81	4.14
3	1	-9	11.52	4.75
-3	1	-9	21.28	3.76
3	-1	-9	15.62	4.49
3	-1	-10	-7.78	3.94
-3	1	-10	-6.31	3.28
3	1	-10	1.19	4.20
3	1	-11	10.62	4.06
3	-1	-11	15.66	3.72
-3	1	-11	13.71	3.31
4	1	0	79.79	8.77
-4	-1	0	71.92	5.37
-4	1	0	64.48	7.45
4	-1	0	78.62	6.34
4	1	0	75.39	4.84
4	1	0	59.81	6.93
-4	1	-1	256.70	8.66
4	1	1	242.76	10.09
4	1	-1	231.09	11.14
-4	-1	-1	264.12	6.88
-4	1	1	241.80	9.12
-4	1	-1	311.39	10.39
-4	-1	1	254.35	7.68
4	1	-1	250.64	8.04
4	-1	1	257.84	7.59
4	1	1	277.57	4.89
4	-1	-1	285.26	9.55
4	1	1	262.33	9.27
4	1	-1	260.60	9.14
-4	1	-2	248.36	8.37
4	-1	-2	241.75	10.37
4	1	-2	254.26	11.14
-4	-1	2	233.05	7.85
4	-1	2	251.42	7.05
-4	-1	-2	245.44	6.18
-4	1	2	234.26	8.94
4	1	-2	236.73	8.71
4	-1	-2	227.87	9.49
4	1	2	254.63	9.08
4	1	2	245.40	8.73
4	1	-2	230.86	8.68
4	1	3	108.45	7.41
-4	1	-3	107.14	6.50
4	-1	3	110.13	4.95
-4	-1	3	108.75	6.62
-4	-1	-3	124.64	4.86
-4	1	3	105.88	7.01
4	1	-3	108.42	7.72
4	-1	-3	121.95	8.21
4	1	-3	114.69	6.81
4	1	-3	98.46	8.64
4	-1	-3	120.33	8.07
-4	1	-4	46.50	5.40
-4	-1	-4	44.35	3.88
4	1	-4	50.74	7.06
-4	-1	4	46.25	5.73
4	-1	4	50.37	3.77
4	1	-4	49.26	7.38
4	-1	-4	39.00	6.34
4	1	4	67.25	6.74
-4	1	4	57.41	5.66
4	-1	-4	48.31	7.12
-4	1	5	93.11	5.54
4	-1	-5	89.07	6.33

-4	-1	-5	98.05	3.94
4	1	-5	102.42	7.75
4	-1	-5	105.01	7.32
-4	-1	5	90.88	5.89
4	-1	5	104.59	3.93
-4	1	-5	98.61	5.69
4	1	-5	92.44	7.19
-4	-1	6	155.18	6.41
-4	1	-6	156.56	5.97
-4	1	6	136.12	5.46
4	1	-6	158.51	8.28
4	-1	-6	152.76	7.55
4	1	-6	152.41	7.60
-4	-1	7	185.19	6.36
4	1	-7	175.89	7.36
-4	1	-7	192.11	6.08
4	1	-7	177.59	8.12
4	-1	-8	8.17	4.93
4	1	-8	10.45	5.18
-4	1	-8	3.62	3.54
4	1	-8	-1.89	4.35
-4	-1	8	7.44	3.48
4	-1	-9	23.38	4.63
-4	-1	9	24.59	3.19
-4	1	-9	29.70	3.66
4	-1	-10	4.27	3.76
-4	1	-10	-2.50	3.13
4	1	-10	-1.05	4.03
5	-1	0	1.98	5.07
5	1	0	1.34	5.27
5	1	0	2.80	3.70
-5	1	0	1.90	5.92
-5	-1	0	3.84	4.18
5	1	0	2.73	7.12
-5	-1	-1	1084.23	12.10
5	1	1	1100.09	14.17
5	1	1	1101.74	16.61
-5	1	1	1099.92	15.35
5	1	-1	1082.47	12.53
5	-1	1	1056.44	12.36
5	1	-1	1113.57	17.78
5	-1	-1	1084.98	14.72
-5	1	-1	1045.62	15.54
-5	-1	1	1069.38	13.39
-5	1	-1	1123.87	14.61
5	1	-1	1090.91	13.51
5	1	2	36.27	5.87
5	-1	-2	40.34	6.68
-5	1	-2	36.30	5.53
-5	-1	-2	45.43	4.13
5	-1	2	45.27	4.36
-5	1	2	47.14	6.27
5	1	-2	42.74	5.70
-5	-1	2	33.89	5.40
5	1	2	54.76	6.84
5	-1	-2	46.63	7.48
5	1	-2	42.42	5.50
5	1	-2	41.19	7.85
5	1	3	163.44	7.46
5	1	-3	168.27	9.20
5	1	-3	172.61	6.74
-5	1	3	159.83	7.53
-5	1	-3	180.59	6.95
-5	-1	-3	187.64	5.78

5	1	-3	167.64	7.89
5	-1	-3	178.07	8.41
-5	-1	3	172.15	7.07
5	-1	3	196.27	5.32
5	-1	-3	161.19	8.36
-5	-1	4	104.31	6.31
5	1	-4	103.45	6.05
5	1	-4	98.42	7.09
-5	1	4	121.18	6.24
-5	-1	-4	113.39	4.95
5	1	-4	103.75	8.00
5	-1	-4	113.25	7.48
5	-1	-4	100.38	6.98
5	-1	4	109.39	4.11
-5	1	-4	120.38	5.87
5	-1	5	35.22	3.06
5	-1	-5	24.53	5.99
-5	-1	-5	33.70	3.45
-5	1	-5	25.75	4.40
-5	1	5	25.99	4.46
5	1	-5	25.14	5.94
5	-1	-5	24.99	5.18
-5	-1	5	29.95	4.92
5	1	-5	29.55	6.37
5	1	-5	30.96	4.61
5	1	-6	0.56	5.30
5	-1	-6	5.69	5.21
-5	-1	6	3.71	4.05
-5	1	6	0.72	3.49
5	1	-6	12.86	5.40
-5	1	-6	5.87	3.87
-5	1	-7	7.79	3.46
5	1	-7	9.52	5.16
5	1	-7	3.33	4.78
-5	-1	7	2.12	3.77
5	-1	-7	10.66	5.04
-5	-1	8	9.12	3.28
-5	1	-8	15.86	3.29
5	1	-8	15.01	4.76
5	-1	-8	10.80	4.47
-5	-1	9	49.27	3.49
-5	1	-9	63.29	4.10
5	1	-9	57.08	5.35
5	-1	-9	65.91	5.08
5	-1	-10	-3.12	3.58
5	1	-10	0.97	3.99
-5	1	-10	-4.20	3.03
-6	1	0	3.22	5.64
6	1	0	-1.69	3.17
6	-1	0	6.49	4.36
6	1	0	1.52	4.56
-6	-1	0	2.35	3.83
6	1	0	-7.29	6.49
6	1	-1	49.93	7.47
-6	-1	-1	47.03	4.29
-6	1	1	38.54	5.79
6	-1	-1	41.06	5.36
6	-1	1	49.16	4.29
-6	1	-1	48.94	6.26
6	1	-1	54.09	4.42
6	1	-1	50.99	5.11
6	1	1	33.72	4.90
-6	1	-1	47.26	5.68
-6	-1	1	43.37	4.82

6	1	1	50.96	6.69
6	1	2	187.30	7.06
-6	-1	-2	184.44	5.75
-6	1	2	198.02	7.61
-6	1	-2	183.79	6.88
6	-1	-2	199.42	7.56
-6	-1	2	203.16	6.82
6	1	-2	211.31	9.26
6	1	-2	195.46	6.47
6	-1	2	187.87	5.55
6	1	-2	181.22	6.26
6	1	-3	-1.26	4.17
6	1	3	9.09	4.66
-6	-1	-3	6.32	3.61
6	1	-3	-0.94	4.62
6	-1	-3	6.18	5.36
-6	1	-3	1.43	4.16
-6	-1	3	2.90	4.41
6	1	-3	5.82	6.19
6	-1	3	7.75	2.93
-6	1	3	-1.82	4.63
6	-1	-3	-2.48	5.73
-6	1	4	3.47	4.17
6	-1	-4	12.19	5.26
6	-1	4	14.91	2.77
6	-1	-4	9.75	5.36
-6	-1	4	8.94	4.41
6	1	-4	10.70	6.02
-6	-1	-4	7.22	3.44
6	1	-4	13.17	4.90
-6	1	-4	12.80	4.28
-6	-1	5	153.64	6.37
6	-1	-5	132.22	6.78
-6	1	5	158.90	6.12
6	1	-5	151.24	7.78
6	1	-5	148.24	7.14
6	-1	-5	157.82	7.31
-6	1	-5	154.57	5.91
-6	-1	6	167.49	6.31
6	1	-6	163.17	7.35
6	-1	-6	168.42	7.36
-6	1	-6	168.21	5.72
6	1	-6	155.50	7.43
6	1	-7	8.24	4.49
6	1	-7	4.56	4.49
-6	-1	7	3.40	3.49
-6	1	-7	11.99	3.58
6	-1	-7	-4.56	4.29
6	1	-8	6.86	4.41
-6	1	-8	7.19	3.11
6	-1	-8	-1.17	4.19
-6	-1	8	0.51	3.00
6	1	-8	-1.44	4.09
-6	1	-9	17.23	3.18
6	1	-9	19.30	4.23
6	-1	-9	6.86	3.88
7	1	0	-5.54	4.02
-7	-1	0	-0.41	3.42
7	-1	0	6.16	3.52
-7	1	0	3.15	4.81
7	1	0	2.27	5.80
-7	1	1	2.11	4.80
7	1	-1	-1.32	3.69
-7	-1	-1	-4.44	3.44

-7	1	-1	-0.23	4.27
7	-1	-1	-0.57	4.07
-7	-1	1	4.57	3.70
7	-1	1	3.40	3.32
7	1	1	7.14	4.13
7	1	-1	2.98	5.86
7	1	1	10.28	5.62
7	1	2	79.82	5.44
7	1	-2	56.78	4.35
7	-1	-2	64.42	5.29
-7	-1	2	56.61	4.58
-7	-1	-2	71.88	4.62
7	1	-2	76.87	7.06
7	-1	2	64.46	3.65
-7	1	-2	74.79	5.44
-7	1	2	63.44	5.35
7	1	-3	17.66	4.08
7	-1	-3	28.81	4.91
7	1	-3	25.99	6.05
-7	-1	3	25.73	4.31
7	1	3	28.91	4.76
-7	1	3	30.54	4.83
-7	-1	-3	23.02	3.79
-7	1	-3	29.28	4.35
7	-1	3	19.73	2.78
-7	-1	-4	59.73	4.06
-7	1	-4	51.38	4.47
-7	1	4	57.22	4.80
-7	-1	-4	65.13	4.57
7	1	-4	58.44	5.04
7	-1	-4	63.31	5.34
7	1	-4	48.44	6.35
7	-1	-4	51.19	5.53
-7	-1	4	66.74	4.97
7	1	-5	37.52	5.91
7	-1	-5	29.95	4.88
-7	1	-5	50.20	4.28
-7	-1	5	33.95	4.19
-7	1	5	32.64	4.16
7	1	-5	42.65	4.94
7	-1	-5	42.06	5.27
-7	1	-6	30.55	3.92
-7	-1	6	21.87	3.88
7	-1	-6	21.26	4.55
-7	1	6	25.45	3.54
7	1	-6	30.49	4.69
7	1	-6	32.77	5.24
7	-1	-7	-2.08	4.00
-7	1	-7	1.22	3.11
7	1	-7	4.61	4.03
7	1	-7	-8.71	4.69
-7	-1	7	-6.38	3.23
-7	-1	8	-2.86	2.91
7	1	-8	3.53	3.79
7	-1	-8	1.88	3.71
-7	1	-8	0.58	2.95
-8	1	0	215.65	6.88
-8	-1	0	231.60	6.26
-8	1	0	237.89	7.60
8	-1	0	208.97	5.91
8	1	0	237.01	8.80
-8	1	-1	28.28	4.32
-8	1	1	21.64	4.46
8	-1	1	27.04	3.10

-8	-1	-1	25.88	3.85
-8	-1	1	30.82	3.85
8	1	-1	14.34	5.62
8	-1	-1	26.98	3.80
-8	1	2	1.09	3.97
-8	-1	-2	-1.55	3.23
-8	1	-2	-0.33	3.59
8	-1	-2	-2.81	3.50
8	1	-2	-5.94	5.42
-8	-1	2	1.76	3.28
-8	-1	3	54.37	4.33
-8	1	3	47.71	4.70
-8	-1	-3	61.32	4.36
8	-1	-3	52.79	4.68
8	1	-3	50.74	5.93
-8	1	-3	49.21	4.46
-8	-1	4	11.06	3.60
8	1	-4	3.48	3.35
8	-1	-4	4.07	3.94
-8	-1	-4	16.18	3.33
-8	1	-4	11.90	3.55
-8	1	4	14.05	3.80
8	1	-4	17.82	5.18
8	1	-5	6.13	3.44
8	-1	-5	-1.45	3.80
-8	1	-5	-0.21	3.28
8	1	-5	0.53	4.68
-8	-1	5	3.46	3.38
-8	1	5	7.14	3.42
-8	-1	6	-3.25	3.15
8	1	-6	5.97	3.63
8	-1	-6	6.58	3.62
-8	1	-6	4.41	3.22
8	1	-7	-2.18	3.28
-8	1	-7	-1.71	3.06
9	-1	0	0.02	2.45
-9	1	0	-0.33	3.37
-9	-1	0	0.57	3.09
-9	-1	-1	1.62	3.20
-9	1	1	7.48	3.60
-9	1	-1	1.72	3.28
9	-1	-1	-4.08	2.86
-9	-1	1	3.55	3.13
9	1	-1	2.18	4.79
-9	1	1	6.96	3.85
-9	1	2	0.86	3.34
-9	1	-2	-0.56	3.23
9	-1	-2	1.70	2.90
-9	-1	2	-3.64	3.05
9	1	-2	-4.26	4.86
-9	1	2	3.06	3.62
-9	-1	-2	4.20	3.13
-9	1	-3	69.76	4.50
-9	1	3	79.56	4.66
-9	-1	-3	87.38	4.43
9	-1	-3	94.17	4.52
-9	-1	3	78.15	4.38
-9	1	3	84.70	4.87
-9	-1	-3	87.53	4.82
-9	-1	4	-5.11	3.22
9	-1	-4	0.43	3.13
-9	-1	-4	-1.17	2.91
-9	1	-4	-0.24	3.16
-9	1	4	2.89	3.30

-9	1	5	8.06	3.15
-9	1	-5	1.46	3.15
-10	-1	0	35.13	3.53
0	2	0	9957.36	45.02
0	-2	0	9994.10	31.99
0	2	0	9999.00	37.76
0	2	1	3.63	6.51
0	2	-1	1.44	6.69
0	-2	-1	2.02	3.96
0	2	-1	3.04	6.00
0	2	1	2.17	4.60
0	-2	1	3.07	4.66
0	2	-2	67.89	7.99
0	2	2	65.03	3.40
0	2	2	80.73	6.76
0	2	-2	75.95	7.35
0	-2	-2	63.44	4.10
0	-2	2	68.32	5.59
0	-2	-3	-0.45	3.41
0	2	3	-1.79	5.44
0	-2	3	4.67	4.67
0	2	-3	3.69	6.84
0	2	-3	7.69	6.89
0	2	-4	628.98	12.98
0	-2	-4	642.23	7.74
0	2	-4	650.48	12.81
0	-2	4	639.87	9.95
0	-2	5	1.75	3.93
0	2	-5	6.35	5.94
0	2	-5	4.02	6.53
0	-2	6	8.00	3.80
0	2	-6	7.55	6.43
0	2	-6	-2.45	5.06
0	2	-7	7.80	5.93
0	-2	7	-4.09	3.24
0	2	-7	2.43	4.55
0	-2	8	94.79	4.14
0	2	-8	89.26	6.46
0	2	-8	85.82	5.47
0	2	-9	-3.33	4.48
0	2	-9	4.16	3.78
0	-2	9	-2.98	2.58
0	2	-10	48.97	4.98
0	2	-10	39.21	4.10
0	2	-11	-3.67	3.74
1	2	0	4.84	6.42
1	2	0	-2.57	8.13
1	-2	0	-0.81	5.22
-1	-2	0	1.01	3.44
-1	2	0	2.72	5.37
-1	2	1	21.24	5.00
-1	2	-1	24.46	6.60
-1	2	1	25.22	4.18
1	-2	-1	22.62	5.24
1	2	-1	19.25	5.62
-1	-2	-1	26.97	2.85
1	-2	1	24.67	5.60
1	2	1	24.69	5.21
-1	-2	1	22.07	4.49
1	2	-1	23.73	7.41
1	2	1	24.30	7.42
1	2	-2	61.45	7.76
-1	2	2	62.61	5.05
-1	2	-2	75.11	7.57

1	-2	2	72.14	5.79
1	2	-2	58.88	7.15
1	-2	-2	74.74	5.84
-1	-2	2	58.81	5.24
1	2	2	66.48	7.39
1	2	2	55.15	4.31
1	2	3	1133.87	14.69
-1	2	3	1130.61	11.71
1	-2	3	1120.78	12.59
-1	-2	3	1081.39	12.58
-1	2	-3	1123.67	15.94
1	2	-3	1121.54	15.96
1	-2	-3	1139.70	12.81
1	2	-3	1133.99	16.22
1	2	4	216.40	8.19
1	2	-4	193.81	9.11
-1	-2	4	199.94	6.63
1	-2	4	208.71	6.70
-1	2	-4	218.67	9.09
1	2	-4	194.66	9.26
1	-2	-4	211.08	6.83
-1	2	-5	215.47	9.15
1	2	-5	212.80	9.03
1	-2	-5	223.31	6.78
1	2	-5	225.91	8.77
1	-2	5	221.12	6.48
-1	-2	5	233.63	6.97
-1	-2	6	305.91	7.09
-1	2	-6	279.80	9.24
1	-2	-6	287.09	7.25
-1	2	-6	286.00	8.22
1	2	-6	309.07	9.10
1	-2	6	295.35	6.48
-1	-2	7	178.08	5.48
-1	2	-7	155.55	6.50
1	2	-7	171.31	7.15
-1	2	-7	163.91	7.52
1	2	-7	162.02	8.23
1	-2	7	167.99	5.18
1	2	-8	4.00	5.47
1	-2	8	2.92	2.62
1	2	-8	-1.40	4.32
-1	-2	8	-3.78	3.14
-1	2	-8	-1.08	3.88
-1	2	-8	11.64	5.01
-1	2	-9	7.10	3.52
1	2	-9	3.61	4.76
1	2	-9	2.98	3.73
-1	-2	9	4.28	2.81
-1	2	-9	10.64	4.36
-1	2	-10	74.56	5.01
1	2	-10	72.76	5.41
1	2	-10	85.17	4.79
-1	2	-11	5.72	3.52
1	2	-11	12.87	3.85
2	2	0	25.89	8.14
2	2	0	34.41	6.92
-2	2	0	36.33	6.04
2	-2	0	37.11	6.10
-2	-2	0	38.87	3.63
2	2	1	2.09	5.95
2	-2	1	11.46	5.53
2	2	-1	11.15	7.85
-2	2	1	6.56	3.17

2	2	-1	2.63	4.70
2	2	1	8.30	7.17
-2	-2	-1	4.13	2.31
-2	-2	1	3.25	3.91
2	-2	-1	8.33	5.80
-2	2	1	2.33	4.84
-2	2	-1	4.50	6.28
2	2	-1	7.48	7.16
2	2	2	325.64	9.92
-2	2	2	318.95	7.04
-2	2	-2	331.15	9.79
2	2	-2	297.81	9.04
2	-2	-2	299.96	8.74
2	2	-2	309.97	10.42
-2	-2	2	334.80	7.37
2	2	2	311.03	7.58
2	-2	2	308.65	8.09
2	2	3	581.89	11.51
-2	2	-3	576.40	11.93
2	2	-3	534.31	11.70
2	-2	-3	582.39	11.33
2	-2	3	566.56	9.64
2	2	-3	574.41	12.66
-2	2	3	546.47	6.97
-2	-2	3	574.12	9.53
2	2	-4	8.50	6.67
2	-2	4	6.73	4.33
2	2	4	4.46	6.12
2	-2	-4	0.07	5.27
2	2	-4	9.95	6.26
-2	-2	4	9.54	4.55
-2	2	-4	9.78	6.01
-2	-2	5	475.02	8.96
2	2	5	496.19	10.96
-2	2	-5	507.49	11.04
2	2	-5	492.01	11.58
2	-2	-5	489.51	9.91
2	2	-5	486.57	11.26
2	-2	5	484.29	8.01
-2	-2	6	252.99	6.74
-2	2	-6	235.66	8.22
2	2	-6	234.85	8.93
2	-2	-6	223.95	7.35
2	2	-6	240.90	8.49
2	-2	6	239.70	5.74
2	-2	-7	8.70	4.30
2	2	-7	3.31	5.65
-2	-2	7	0.51	3.67
2	-2	7	1.89	2.76
-2	2	-7	5.09	5.07
-2	2	-7	3.28	4.04
2	2	-7	3.85	4.72
-2	-2	8	55.60	4.15
-2	2	-8	54.28	5.17
2	2	-8	53.39	6.08
2	-2	-8	49.90	4.74
-2	2	-8	48.16	4.32
2	-2	8	53.14	3.26
2	2	-8	55.42	5.14
-2	-2	9	15.54	3.08
-2	2	-9	9.44	3.30
2	2	-9	8.99	3.98
-2	2	-9	15.23	4.22
2	2	-9	8.77	4.93

-2	2	-10	4.36	3.51
2	2	-10	6.39	3.63
2	2	-10	-1.87	4.20
3	2	0	-2.07	7.18
3	-2	0	-0.73	5.42
-3	2	0	-0.35	5.20
-3	-2	0	-0.56	3.19
3	2	0	10.12	6.86
-3	-2	1	1071.36	11.32
-3	-2	-1	1095.43	8.57
3	2	1	1091.26	14.96
3	2	-1	1139.05	15.97
-3	2	1	1102.67	12.98
-3	2	-1	1090.87	14.43
3	2	-1	1055.97	11.24
3	-2	-1	1055.84	14.13
3	-2	1	1095.27	13.59
3	2	-1	1140.72	16.44
3	2	1	1103.95	15.48
-3	-2	2	985.25	11.42
-3	-2	-2	981.65	7.05
-3	2	2	983.51	11.63
3	2	-2	974.01	12.92
3	-2	-2	983.17	13.95
3	2	-2	996.58	15.93
-3	2	-2	971.06	14.04
3	-2	2	977.79	12.37
3	2	-2	1008.61	15.38
3	2	2	989.13	12.72
3	2	2	944.23	14.69
-3	2	3	117.08	5.17
3	2	3	111.07	7.45
-3	-2	3	121.94	5.71
3	2	-3	105.94	8.27
3	2	3	119.70	5.36
-3	2	-3	103.60	7.08
3	-2	3	134.91	5.95
3	2	-3	96.04	7.20
3	-2	-3	117.82	7.10
3	2	-4	-2.21	6.24
3	2	-4	7.07	6.06
3	-2	4	1.22	3.90
-3	-2	4	3.75	4.19
-3	2	-4	-2.10	5.61
3	2	4	3.81	5.82
3	-2	-4	4.20	5.74
3	2	-5	0.75	5.84
3	2	5	3.49	5.54
3	-2	-5	4.87	5.16
3	-2	5	0.98	3.39
-3	2	-5	-1.93	5.29
-3	-2	5	1.15	4.21
3	2	-5	2.47	5.74
3	-2	-6	6.48	4.72
3	-2	6	8.07	3.02
3	2	-6	11.21	5.72
-3	2	-6	7.40	4.80
3	2	-6	10.52	5.28
-3	-2	6	7.62	3.97
3	2	-7	18.88	5.73
-3	-2	7	24.10	4.28
3	-2	-7	21.52	4.72
-3	2	-7	24.21	4.69
3	2	-7	27.21	5.14

3	-2	7	24.97	2.86
3	2	-8	6.59	5.12
-3	-2	8	7.00	3.48
3	-2	-8	3.24	4.05
-3	2	-8	3.12	4.00
-3	2	-8	3.73	3.39
3	2	-8	9.02	4.47
-3	-2	9	122.02	4.76
-3	2	-9	118.34	5.43
3	2	-9	128.79	6.85
3	-2	-9	128.35	5.74
-3	2	-9	108.49	4.81
3	2	-9	118.46	6.00
3	2	-10	11.82	4.56
-3	2	-10	5.02	3.23
-4	-2	0	29.42	3.76
4	-2	0	32.29	5.52
-4	2	0	32.80	5.52
4	2	0	40.80	7.16
4	2	0	28.11	6.47
-4	2	-1	196.12	7.62
-4	-2	1	193.35	5.93
-4	-2	-1	189.42	5.02
-4	2	1	198.85	7.39
4	2	-1	199.61	5.51
4	-2	-1	191.20	7.83
4	-2	1	193.62	7.04
4	2	1	221.22	8.60
4	2	-1	203.05	8.88
4	2	-1	216.70	9.28
4	2	1	199.62	8.34
4	2	2	345.64	9.95
-4	2	2	335.38	8.49
-4	-2	2	349.55	7.70
-4	-2	-2	344.57	5.71
4	-2	2	343.93	8.02
-4	2	-2	346.70	9.05
4	2	-2	344.61	8.04
4	-2	-2	339.74	9.51
4	2	-2	319.02	10.50
4	2	2	352.93	9.08
4	2	3	30.45	5.98
4	-2	3	44.74	4.55
-4	-2	3	36.02	4.57
-4	2	3	37.57	4.75
-4	2	-3	46.84	6.01
4	2	-3	31.00	5.50
4	-2	-3	38.88	6.11
4	2	-3	44.37	7.07
4	2	3	40.21	4.92
4	2	-3	33.16	6.32
4	2	4	171.32	7.61
-4	-2	4	165.96	6.45
-4	2	4	167.65	5.86
-4	2	-4	166.43	7.09
4	2	-4	183.07	8.43
4	2	-4	155.35	7.59
4	-2	-4	186.59	7.99
4	-2	4	170.26	5.27
4	2	-4	182.09	7.40
4	-2	5	3.90	2.99
4	2	-5	4.20	5.31
4	2	5	0.63	5.22
4	-2	-5	3.27	4.98

-4	2	-5	-0.64	4.68
4	2	-5	0.75	5.43
-4	-2	5	9.63	4.36
-4	-2	6	203.39	6.77
-4	2	-6	216.33	7.07
4	2	-6	216.20	8.13
4	-2	-6	202.79	7.60
4	-2	6	212.42	5.15
4	2	-6	211.97	8.02
4	2	-7	34.38	5.51
4	2	-7	60.06	5.55
-4	-2	7	52.18	4.58
-4	2	-7	55.64	4.99
4	-2	-7	59.38	5.28
4	-2	7	43.07	2.99
-4	-2	8	69.94	4.62
-4	2	-8	70.04	4.74
4	2	-8	72.02	6.17
4	-2	-8	58.56	5.05
4	2	-8	82.06	5.67
-4	2	-9	0.80	3.03
4	2	-9	2.55	4.54
-4	-2	9	10.71	3.09
4	-2	-9	4.96	3.67
-4	2	-9	6.85	3.57
4	2	-10	-2.04	4.20
4	-2	-10	-0.18	3.38
-4	2	-10	0.40	3.16
5	2	0	5.45	5.40
-5	-2	0	5.14	3.45
-5	2	0	-1.30	4.91
5	-2	0	7.54	4.78
5	2	0	-3.70	6.10
-5	-2	1	41.54	4.36
5	-2	1	58.49	5.08
-5	-2	-1	49.30	3.72
-5	2	1	48.51	5.80
-5	2	-1	47.12	5.73
5	-2	-1	53.17	5.68
5	2	-1	53.29	7.19
5	2	1	48.82	6.34
5	2	-1	55.38	6.02
5	2	1	57.44	6.39
-5	-2	2	76.51	5.08
5	2	-2	67.03	7.36
-5	2	2	86.73	5.81
-5	-2	-2	78.56	3.92
-5	2	-2	71.20	6.01
5	2	-2	78.52	4.83
5	2	2	84.93	6.32
5	-2	2	85.87	4.98
5	2	2	66.88	6.16
5	-2	-2	78.93	6.34
5	2	-2	96.30	6.46
5	-2	-3	32.02	5.63
5	2	3	24.05	5.45
5	2	-3	11.60	5.43
-5	-2	3	21.05	4.39
-5	2	-3	27.71	5.39
-5	2	3	25.41	4.54
5	2	-3	25.84	4.69
5	2	-3	31.78	6.61
5	-2	3	23.06	3.73
5	2	4	419.42	9.62

-5	-2	4	449.02	9.07
-5	2	4	431.38	8.42
-5	2	-4	445.32	9.34
5	2	-4	437.93	9.96
5	2	-4	449.58	9.69
5	-2	-4	469.75	10.45
5	2	-4	447.77	11.28
5	-2	4	427.98	7.17
5	2	-5	8.51	4.92
-5	-2	5	-0.48	3.78
5	2	-5	-0.50	4.72
5	2	5	12.74	4.92
5	-2	-5	12.62	5.24
5	-2	5	3.11	2.73
-5	2	-5	-1.34	4.25
5	2	-5	1.32	5.39
5	2	-6	4.39	4.19
5	2	-6	-5.64	4.85
-5	-2	6	2.57	3.83
5	-2	6	3.79	2.40
5	-2	-6	0.67	4.63
-5	2	-6	1.43	3.91
5	2	-6	4.39	5.19
5	2	-7	2.52	4.62
-5	-2	7	-2.11	3.59
5	-2	-7	-0.26	4.08
5	2	-7	1.64	3.74
-5	2	-7	1.03	3.71
5	2	-7	-0.68	4.86
5	-2	-8	13.16	4.03
-5	-2	8	18.22	3.69
5	2	-8	28.45	4.83
5	2	-8	25.36	4.89
-5	2	-8	16.51	3.54
-5	2	-9	18.62	3.38
-5	-2	9	20.06	3.30
5	2	-9	21.67	4.79
5	-2	-9	22.23	4.00
-6	-2	0	29.49	3.79
-6	2	0	28.79	5.19
6	-2	0	21.91	4.28
6	2	0	28.87	6.07
6	2	0	23.86	5.02
6	2	1	198.11	7.36
6	2	-1	185.24	8.58
-6	-2	1	195.69	6.06
-6	2	1	188.01	7.26
-6	2	-1	181.17	7.19
-6	-2	-1	206.74	5.74
6	-2	-1	184.84	6.88
6	-2	1	186.74	6.12
6	2	1	189.46	7.73
6	2	-1	173.79	6.77
6	2	-2	11.25	4.70
6	2	2	4.63	4.53
-6	-2	2	6.73	3.60
6	2	-2	5.92	5.84
6	-2	-2	2.90	4.57
-6	2	2	4.90	4.56
-6	2	-2	7.02	4.72
6	-2	2	6.39	3.68
-6	-2	-2	8.24	3.12
6	2	2	8.55	5.06
6	2	3	89.89	5.95

6	-2	-3	84.74	6.13
-6	-2	3	71.49	5.07
-6	2	3	63.95	5.09
-6	2	-3	82.33	5.68
6	2	-3	78.04	4.72
6	2	-3	71.48	6.92
6	-2	3	78.50	3.99
6	2	-3	76.85	5.60
6	2	-4	11.59	4.52
6	2	4	17.41	4.79
-6	-2	4	15.47	4.14
6	2	-4	11.71	4.14
6	-2	-4	2.49	4.50
-6	2	4	10.33	3.78
-6	2	-4	13.12	4.43
6	-2	4	19.34	2.98
6	2	-4	13.46	5.72
6	-2	5	1.10	2.49
6	2	-5	-4.98	4.39
6	2	-5	4.09	4.17
-6	-2	5	3.47	3.79
6	-2	-5	0.95	4.58
-6	2	-5	0.26	3.95
6	2	-5	-0.72	5.13
6	2	-6	2.40	4.19
6	2	-6	-4.96	4.34
6	-2	-6	2.83	4.37
-6	-2	6	-0.42	3.77
-6	2	-6	-0.16	3.60
6	2	-6	2.18	4.78
-6	-2	7	37.84	4.39
-6	2	-7	39.37	4.16
6	2	-7	35.59	4.85
6	-2	-7	30.54	4.52
6	2	-7	36.61	5.24
-6	2	-8	52.61	4.04
6	-2	-8	45.02	4.65
7	2	0	2.60	5.24
7	2	0	-7.59	4.24
-7	2	0	-1.43	4.58
7	-2	0	-6.92	3.73
-7	-2	0	-0.67	3.06
7	2	1	51.22	4.86
-7	2	1	56.31	5.20
-7	2	-1	67.69	5.45
-7	-2	1	53.92	4.22
-7	-2	-1	47.70	3.80
7	-2	-1	43.08	4.69
7	-2	1	57.47	4.16
7	2	-1	53.87	6.28
7	2	1	66.81	6.05
7	2	-1	46.64	4.81
7	2	2	146.72	6.24
-7	2	2	137.43	6.27
-7	-2	2	131.25	5.42
-7	2	-2	142.56	6.21
-7	-2	-2	132.80	4.75
7	-2	-2	133.97	6.05
7	2	-2	114.86	7.51
7	-2	2	124.26	4.64
7	2	2	130.42	6.43
7	2	-2	121.05	5.61
7	2	3	30.46	4.52
-7	2	3	22.01	4.34

-7	-2	3	28.15	3.91
-7	2	-3	34.63	4.73
7	-2	-3	24.93	4.49
7	-2	3	30.48	3.12
7	2	-3	35.41	5.85
7	2	-3	31.21	4.06
7	2	4	43.79	4.72
-7	2	4	36.19	4.10
-7	-2	4	46.03	4.32
-7	2	-4	37.67	4.44
7	-2	-4	42.68	4.88
7	2	-4	48.21	5.97
7	-2	4	36.35	2.99
7	-2	-5	10.58	4.13
-7	-2	5	8.81	3.50
-7	2	-5	10.62	3.71
7	2	-5	5.75	4.75
7	2	-6	3.90	3.66
7	-2	-6	15.94	3.97
-7	-2	6	7.75	3.57
-7	2	-6	8.13	3.31
7	2	-6	-0.12	4.52
-7	-2	7	41.70	4.08
-7	2	-7	40.32	3.93
7	2	-7	36.65	4.32
7	-2	-7	43.02	4.54
8	2	0	77.64	4.80
-8	2	0	82.17	5.20
-8	-2	0	86.28	4.30
8	2	0	90.50	6.12
8	-2	0	79.92	4.35
8	2	-1	2.94	3.51
8	2	1	4.22	3.76
-8	-2	-1	2.40	2.84
-8	2	1	12.92	4.18
-8	2	-1	11.88	4.11
8	-2	-1	3.53	3.30
8	-2	1	5.53	2.85
8	2	-1	5.52	4.89
8	2	1	13.26	4.84
-8	-2	1	5.73	3.09
8	2	2	17.01	4.12
-8	2	-2	18.08	4.20
-8	2	2	12.29	3.98
8	-2	-2	8.17	3.65
-8	-2	-2	12.94	2.97
-8	-2	2	7.40	3.15
8	-2	2	16.01	2.76
8	2	-2	14.16	5.22
8	2	3	32.16	4.30
-8	2	3	24.08	3.88
-8	2	-3	22.79	4.15
-8	-2	3	22.85	3.55
8	2	-3	21.39	5.45
8	-2	-3	36.98	4.28
8	-2	3	24.69	2.68
-8	-2	4	15.47	3.47
8	-2	-4	8.62	3.74
-8	2	4	1.94	3.26
-8	2	-4	12.41	3.66
8	2	-4	7.40	4.91
-8	-2	5	62.69	4.33
-8	2	-5	63.14	4.22
8	-2	-5	58.89	4.79

8	-2	-6	-3.53	3.58
-8	2	-6	7.49	3.27
-8	-2	6	0.86	3.15
-9	-2	0	2.97	2.81
-9	2	0	-2.30	3.41
9	-2	0	-0.14	2.64
9	-2	1	0.77	2.43
-9	-2	1	6.17	2.78
-9	2	1	0.49	3.36
-9	2	-1	0.92	3.37
9	-2	-1	0.48	2.74
-9	-2	-1	4.16	2.78
-9	2	2	31.20	3.73
-9	2	-2	29.45	4.08
-9	-2	2	37.38	3.47
9	-2	-2	29.16	3.55
-9	-2	3	7.78	3.01
-9	2	3	9.98	3.37
9	-2	-3	-0.18	3.07
-9	2	-3	0.28	3.39
0	3	0	4.29	4.70
0	3	0	-2.90	6.49
0	-3	0	-0.46	3.40
0	3	1	-0.57	5.27
0	-3	-1	-1.03	3.22
0	3	-1	-0.62	5.17
0	3	1	3.41	3.98
0	-3	1	2.02	3.75
0	3	-1	6.57	4.21
0	3	-1	10.44	6.91
0	3	2	0.66	4.61
0	-3	-2	-1.60	2.95
0	3	-2	0.51	5.65
0	3	-2	-2.72	4.91
0	-3	2	2.46	3.77
0	3	-3	674.67	11.68
0	3	-3	700.10	12.48
0	3	3	687.62	10.39
0	-3	3	687.85	9.69
0	3	4	4.82	4.38
0	-3	4	1.47	3.64
0	3	-4	5.08	5.84
0	3	-4	7.78	5.49
0	3	-5	390.64	9.95
0	-3	5	382.28	7.69
0	-3	6	0.85	3.41
0	3	-6	5.11	5.30
0	3	-6	-4.87	4.96
0	3	-7	70.21	6.31
0	3	-7	81.59	5.75
0	-3	7	76.42	4.36
0	-3	8	-1.79	3.04
0	3	-8	0.73	4.27
0	3	-8	5.73	4.60
0	-3	9	1.34	2.60
0	3	-9	-2.04	3.72
0	3	-9	1.50	4.48
0	3	-10	-4.13	3.96
-1	3	0	2.03	5.73
1	3	0	-1.29	6.79
1	-3	0	-0.03	4.03
-1	3	0	2.63	3.81
1	3	0	6.03	5.14
-1	-3	0	-0.55	2.98

1	-3	-1	35.87	4.27
-1	3	-1	29.43	4.90
1	3	1	40.45	4.57
1	3	-1	36.16	5.79
-1	-3	-1	35.89	2.94
-1	-3	1	34.60	3.85
1	3	-1	39.18	7.61
-1	3	1	30.34	4.71
1	3	1	31.17	6.33
1	-3	1	33.53	4.14
1	-3	-2	719.26	9.35
-1	3	-2	724.98	11.27
1	3	-2	717.12	10.49
1	3	-2	714.32	12.46
1	-3	2	699.38	9.81
-1	3	2	708.89	8.16
-1	-3	2	704.76	9.22
-1	3	2	694.40	9.49
1	3	3	250.06	7.69
-1	3	3	233.25	5.97
-1	3	-3	232.29	7.93
1	3	-3	240.37	7.75
1	-3	-3	232.63	6.03
1	3	-3	239.74	8.84
1	-3	3	248.01	6.61
-1	-3	3	247.57	6.43
1	3	-4	8.33	5.24
-1	3	4	14.66	3.77
1	-3	4	4.46	3.89
1	3	4	5.67	4.78
-1	-3	4	11.63	3.91
-1	3	-4	6.29	5.52
-1	3	-4	7.29	5.65
1	3	-4	6.72	6.01
1	3	5	6.50	4.87
1	-3	5	-6.82	3.55
-1	-3	5	3.44	3.69
-1	3	-5	-0.29	5.17
-1	3	-5	-3.99	5.33
1	3	-5	4.85	5.69
1	3	-5	-6.42	5.33
-1	-3	6	21.53	3.88
-1	3	-6	27.83	5.73
-1	3	-6	27.74	5.24
1	3	-6	22.64	5.21
1	3	-6	24.37	5.56
1	-3	6	22.44	3.66
-1	-3	7	137.66	5.08
1	3	-7	140.64	6.97
-1	3	-7	144.35	7.10
-1	3	-7	136.29	6.35
1	-3	7	133.58	4.80
1	-3	8	34.35	3.34
-1	-3	8	30.58	3.55
-1	3	-8	22.59	5.14
1	3	-8	39.47	5.40
1	3	-8	34.46	4.98
-1	3	-8	29.05	4.24
1	-3	9	34.17	2.95
-1	-3	9	35.74	3.39
-1	3	-9	44.23	5.04
1	3	-9	33.33	5.14
-1	3	-9	34.25	4.19
1	3	-9	42.91	4.62

-1	-3	10	44.88	3.24
-1	3	-10	54.97	4.88
1	3	-10	44.93	5.05
-2	3	0	780.19	10.46
2	3	0	767.68	11.94
2	-3	0	772.13	10.62
-2	-3	0	764.07	8.39
-2	3	0	750.04	10.35
-2	3	-1	609.75	10.15
2	3	1	609.37	9.58
2	3	-1	647.89	11.71
2	-3	-1	595.76	9.53
2	-3	1	622.70	9.89
-2	-3	-1	617.02	6.50
-2	-3	1	596.90	8.23
2	3	-1	610.10	13.03
2	3	1	635.56	11.98
-2	3	-2	104.54	6.41
2	3	-2	96.98	5.20
2	3	-2	99.36	7.11
2	-3	-2	100.85	5.48
2	3	2	89.09	4.77
2	-3	2	91.62	5.26
2	3	-2	90.32	7.89
-2	-3	2	98.28	4.74
2	3	2	92.89	6.67
2	3	3	102.01	6.47
-2	3	-3	98.35	6.43
2	3	-3	109.92	6.21
2	3	-3	118.43	7.36
2	-3	-3	106.94	5.53
2	-3	3	95.68	5.31
-2	-3	3	101.17	4.98
2	-3	4	9.68	4.02
2	3	4	6.52	4.95
2	-3	-4	10.17	4.25
-2	-3	4	2.31	3.61
-2	3	-4	3.77	5.18
2	3	-4	-4.23	5.69
2	3	-4	5.51	4.93
-2	-3	5	155.41	5.68
2	3	5	136.29	6.44
-2	3	-5	140.49	6.91
2	3	-5	141.16	6.95
2	-3	-5	131.71	5.81
2	3	-5	127.85	7.48
2	-3	5	138.68	5.27
-2	-3	6	17.38	3.84
2	3	-6	21.24	5.44
-2	3	-6	17.49	5.25
-2	3	-6	15.88	4.61
2	-3	6	18.04	3.41
2	3	-6	22.38	5.28
2	-3	7	13.11	3.14
-2	3	-7	17.39	4.25
-2	3	-7	17.55	4.93
-2	-3	7	9.06	3.42
2	3	-7	14.69	4.86
2	3	-7	11.64	5.02
-2	-3	8	83.44	4.54
-2	3	-8	99.55	5.82
2	3	-8	95.98	6.14
-2	3	-8	99.45	5.18
2	3	-8	83.26	5.65

2	-3	8	91.49	3.86
2	3	-9	-4.60	4.38
2	-3	9	0.35	2.22
-2	3	-9	-0.01	3.27
2	3	-9	2.14	3.94
-2	3	-9	0.03	3.93
-2	-3	9	2.27	2.81
2	3	-10	16.18	4.22
-2	-3	10	21.44	2.84
-2	3	-10	17.45	3.88
3	3	0	5.20	6.56
-3	-3	0	4.08	2.62
3	-3	0	5.33	4.27
-3	3	0	-2.56	4.40
3	3	0	3.17	5.09
-3	3	1	102.98	5.25
-3	3	-1	77.76	5.65
3	3	-1	110.94	7.13
3	3	1	87.02	5.85
3	-3	1	95.15	5.41
3	-3	-1	89.23	5.55
3	3	-1	90.68	7.84
3	3	1	96.09	6.88
-3	-3	1	102.86	4.31
3	3	-2	1.69	6.54
3	3	2	8.75	5.74
3	-3	-2	-0.01	4.51
-3	-3	2	5.14	3.41
-3	3	-2	3.72	4.89
3	-3	2	-1.14	4.01
3	3	-2	-1.17	5.86
3	3	2	2.48	4.15
3	3	3	22.62	5.55
-3	-3	3	15.49	3.77
3	3	-3	21.32	4.47
3	-3	-3	24.78	4.70
-3	3	-3	19.60	5.22
3	-3	3	25.87	4.35
3	3	-3	13.08	5.90
3	3	-3	18.47	6.13
3	3	4	449.35	9.57
-3	-3	4	460.57	8.35
3	3	-4	454.05	10.93
-3	3	-4	434.75	9.69
3	-3	-4	445.72	9.27
3	-3	4	442.32	8.09
3	3	5	100.52	6.20
-3	-3	5	100.45	4.93
-3	3	-5	102.23	6.21
3	3	-5	91.70	6.24
3	-3	-5	112.48	5.80
3	3	-5	102.12	7.04
3	-3	5	102.79	4.72
-3	-3	6	34.80	4.08
-3	3	-6	38.92	5.07
3	3	-6	31.10	5.13
3	-3	-6	26.77	4.34
3	3	-6	24.01	5.26
3	-3	6	27.36	3.33
3	3	-7	8.16	4.80
3	-3	7	3.90	2.60
-3	3	-7	6.09	4.52
3	3	-7	7.69	4.87
-3	-3	7	4.05	3.47

-3	-3	8	26.95	3.64
-3	3	-8	28.49	4.59
3	3	-8	26.06	4.96
-3	3	-8	24.57	3.93
3	3	-8	21.06	4.62
3	-3	8	29.78	2.70
-3	-3	9	11.64	3.07
-3	3	-9	10.87	3.88
-4	-3	0	16.00	2.67
4	3	0	22.91	5.89
-4	3	0	15.56	4.31
4	-3	0	20.64	4.29
4	3	0	16.67	5.37
-4	-3	1	54.72	3.58
4	3	-1	67.69	6.40
-4	3	1	57.01	4.64
-4	3	-1	59.32	5.24
4	-3	-1	62.76	5.10
4	3	1	78.83	5.92
4	-3	1	67.34	4.99
4	3	-1	58.39	6.38
4	3	1	64.83	6.43
-4	-3	2	65.42	4.07
-4	3	-2	61.27	5.30
4	-3	-2	67.52	5.27
4	3	-2	80.37	6.76
4	3	2	66.94	5.02
4	-3	2	79.28	4.81
4	3	-2	74.69	6.63
4	3	2	75.64	6.45
4	3	3	34.23	5.49
-4	-3	3	35.47	3.83
-4	3	-3	30.56	4.97
4	3	-3	37.77	5.98
4	-3	3	40.21	4.31
4	3	-3	40.40	6.23
4	-3	-3	35.40	5.00
-4	-3	4	20.07	3.88
4	3	-4	13.99	4.20
4	3	4	10.45	4.78
4	-3	-4	20.66	4.62
-4	3	-4	10.72	4.69
4	3	-4	16.30	5.45
4	3	-4	25.61	5.76
4	-3	4	14.80	3.56
4	3	5	43.58	5.34
-4	-3	5	46.28	4.15
-4	3	-5	40.48	5.08
4	3	-5	37.63	5.04
4	-3	-5	36.91	4.73
4	3	-5	43.89	5.87
4	-3	5	43.61	3.66
4	3	-5	56.26	5.90
-4	-3	6	81.44	4.70
-4	3	-6	64.58	5.29
4	3	-6	63.97	5.40
4	-3	-6	55.97	4.91
4	3	-6	57.43	5.86
4	-3	6	74.46	3.81
-4	-3	7	76.39	4.79
-4	3	-7	97.95	5.49
4	3	-7	85.67	5.81
4	-3	-7	82.40	5.06
4	3	-7	79.03	5.75

4	-3	7	92.81	3.83
-4	-3	8	3.06	3.26
4	3	-8	-4.90	4.15
-4	3	-8	3.17	3.73
-4	3	-8	7.66	3.35
4	3	-8	-4.76	4.33
4	3	-9	18.44	4.24
-4	-3	9	13.81	3.16
-4	3	-9	19.12	3.66
5	3	0	-3.81	5.13
-5	3	0	-2.10	4.15
5	-3	0	4.67	4.02
5	3	0	-5.21	5.28
-5	-3	0	1.85	2.70
-5	-3	1	379.33	6.89
-5	3	1	403.17	8.29
-5	3	-1	389.62	8.53
5	-3	-1	398.35	8.53
5	3	-1	393.48	10.15
5	3	1	411.21	9.38
5	-3	1	375.94	7.86
5	3	-1	393.91	9.48
5	3	1	397.99	9.49
5	3	2	22.74	5.26
5	3	-2	6.99	5.18
-5	-3	2	13.34	3.31
-5	3	-2	18.23	4.63
-5	3	2	7.27	3.83
5	-3	-2	20.87	4.42
5	-3	2	20.04	3.81
5	3	-2	23.70	6.00
5	3	3	70.51	5.56
-5	-3	3	68.44	4.42
-5	3	-3	63.97	5.17
5	-3	-3	64.78	5.23
5	3	-3	72.08	6.44
5	-3	3	66.08	4.26
5	3	-3	62.15	6.12
5	3	4	45.73	5.07
-5	-3	4	45.45	4.31
-5	3	-4	46.98	4.88
5	-3	-4	38.77	4.96
5	3	-4	51.13	6.07
5	-3	4	41.64	3.55
5	3	-4	48.19	5.80
-5	-3	5	13.89	3.68
5	-3	5	14.61	2.95
5	3	5	16.68	4.65
5	3	-5	12.34	5.14
5	3	-5	17.68	5.51
5	-3	-5	13.91	4.45
-5	3	-5	13.37	4.32
-5	-3	6	1.65	3.44
5	3	-6	7.67	4.64
5	-3	6	5.28	2.47
5	-3	-6	-3.48	3.84
-5	3	-6	0.67	3.97
5	3	-6	7.79	5.02
5	3	-7	5.72	4.12
-5	-3	7	-4.83	3.38
5	3	-7	2.26	3.92
5	-3	7	-2.63	2.20
5	-3	-7	0.38	3.41
-5	3	-7	2.85	3.66

5	3	-7	3.16	4.47
5	3	-8	7.10	3.93
5	3	-8	7.64	3.84
-5	-3	8	4.42	3.16
5	-3	-8	9.52	3.43
-5	3	-8	0.44	3.58
6	3	0	3.20	4.57
-6	-3	0	0.42	2.74
-6	3	0	-1.90	4.15
6	-3	0	4.80	3.51
6	3	0	-1.03	4.94
6	3	1	18.98	4.94
6	-3	-1	21.73	4.06
-6	-3	1	17.37	3.13
-6	3	-1	13.77	4.49
-6	3	1	5.48	3.98
6	-3	1	19.32	3.75
6	3	-1	22.67	5.34
6	3	1	15.73	4.77
6	3	-1	22.17	4.79
6	3	2	83.28	5.69
-6	-3	2	89.93	4.47
-6	3	2	77.35	4.99
-6	3	-2	79.77	5.29
6	-3	-2	90.79	5.16
6	3	-2	84.10	6.72
6	-3	2	77.17	4.39
6	3	-2	87.93	5.62
6	3	-3	1.91	4.53
-6	-3	3	0.18	3.12
6	3	3	8.11	4.51
6	-3	-3	8.90	4.00
-6	3	-3	4.16	4.10
6	-3	3	2.41	2.92
6	3	-3	3.14	5.24
6	3	-4	5.03	4.42
-6	-3	4	4.67	3.42
6	3	4	3.47	4.23
6	-3	-4	8.82	4.12
-6	3	-4	10.38	3.93
6	3	-4	2.25	5.18
6	-3	4	2.22	2.65
6	3	5	88.17	5.53
-6	-3	5	80.66	4.73
-6	3	-5	72.68	5.05
6	-3	-5	72.13	5.05
6	3	-5	71.67	6.46
6	-3	5	73.25	3.50
6	3	-5	68.01	5.41
-6	-3	6	79.05	4.65
-6	3	-6	89.36	5.03
6	-3	-6	91.32	5.14
6	3	-6	83.19	6.30
6	-3	6	82.96	3.38
6	3	-6	76.77	5.28
6	3	-7	-2.19	3.86
-6	-3	7	6.58	3.26
6	-3	-7	2.54	3.21
-6	3	-7	8.31	3.51
7	3	0	-0.84	4.19
-7	-3	0	2.83	2.93
-7	3	0	-3.21	4.02
7	-3	0	0.48	3.22
7	3	0	-6.90	4.61

7	3	-1	2.12	3.95
7	3	1	1.40	3.95
-7	-3	1	2.48	2.99
-7	3	1	-0.03	3.83
-7	3	-1	-1.10	4.00
7	-3	-1	1.06	3.46
7	-3	1	-0.99	3.11
7	3	-1	-4.57	4.89
7	3	1	-4.22	4.23
7	3	2	33.06	4.49
-7	-3	2	31.79	3.65
-7	3	-2	35.12	4.61
7	-3	-2	42.33	4.34
7	3	-2	33.35	5.46
7	-3	2	31.17	3.30
7	3	-2	33.07	4.44
7	3	-3	14.21	4.11
7	3	3	19.71	4.15
-7	-3	3	11.85	3.17
-7	3	-3	18.51	4.12
7	-3	-3	21.11	3.99
7	-3	3	13.71	2.82
7	3	4	37.60	4.45
-7	-3	4	24.69	3.55
-7	3	-4	28.86	4.25
7	3	-4	34.38	5.46
7	-3	-4	33.57	4.32
7	-3	4	33.00	2.97
7	3	-4	28.75	4.36
7	3	5	24.07	4.25
7	3	-5	13.21	4.02
-7	-3	5	25.52	3.73
-7	3	-5	27.43	4.05
7	-3	-5	23.91	3.88
7	-3	5	17.28	2.40
-7	3	-6	18.93	3.88
7	-3	-6	13.35	3.61
8	3	0	118.52	5.39
-8	3	0	127.23	5.69
8	-3	0	129.77	4.91
8	3	0	122.58	6.36
8	3	-1	11.74	3.85
8	3	1	11.92	3.70
-8	-3	1	12.89	2.90
-8	3	1	19.18	3.85
-8	3	-1	3.35	3.84
8	-3	-1	12.91	3.25
8	-3	1	18.29	2.98
8	3	-1	10.65	4.60
8	3	-2	1.44	3.58
8	3	2	2.86	3.61
-8	3	-2	-0.77	3.67
8	-3	-2	0.17	3.16
-8	-3	2	-0.66	2.74
8	3	3	25.12	4.03
-8	-3	3	23.40	3.28
-8	3	-3	36.26	4.10
8	-3	-3	30.92	3.79
8	-3	3	30.57	2.83
8	3	-3	26.38	3.98
8	3	4	13.94	3.80
8	-3	-4	14.26	3.33
-8	-3	4	5.94	2.86
-8	3	-4	11.57	3.71

8	-3	4	11.25	2.27
0	4	0	1361.76	13.94
0	-4	0	1353.22	11.84
0	4	0	1376.91	16.76
0	4	-1	-0.10	6.30
0	4	1	4.91	4.51
0	4	-1	-4.31	4.43
0	-4	-1	-2.54	2.67
0	-4	1	-0.59	3.07
0	-4	2	14.77	3.28
0	4	-2	19.20	6.27
0	4	2	10.53	3.96
0	4	-2	8.35	4.62
0	4	3	0.34	3.64
0	-4	3	2.90	3.18
0	4	-3	-7.53	5.20
0	4	-4	130.43	5.92
0	4	-4	124.75	6.59
0	-4	4	134.38	4.87
0	-4	5	-1.81	3.05
0	4	-5	-7.27	5.11
0	4	-5	1.19	4.10
0	-4	6	1.54	2.87
0	4	-6	-1.48	4.95
0	4	-6	-6.46	4.41
0	-4	7	3.41	2.82
0	4	-7	2.27	4.41
0	4	-7	-1.60	4.19
0	-4	8	31.41	3.24
0	4	-8	32.37	4.69
0	-4	9	3.65	2.53
0	4	-9	7.72	3.79
-1	4	0	-1.68	4.81
1	4	0	-7.17	6.30
-1	4	0	0.67	4.02
1	-4	0	-0.69	3.05
1	4	0	-3.56	4.21
-1	-4	0	0.51	2.74
-1	4	-1	7.04	5.77
1	4	-1	0.87	6.61
-1	-4	1	4.02	2.91
1	4	1	6.60	5.11
1	-4	-1	7.68	3.23
-1	4	-1	5.08	4.23
1	4	-1	4.60	4.54
1	-4	1	4.47	3.35
-1	4	-2	11.69	5.58
1	4	-2	1.23	6.26
1	4	2	5.05	4.33
-1	-4	2	7.01	3.12
1	-4	-2	2.74	2.90
-1	4	-2	5.50	4.46
1	4	-2	4.87	4.67
1	-4	2	9.34	3.28
1	4	3	168.90	6.34
-1	4	-3	194.56	6.62
-1	4	-3	178.18	6.96
1	4	-3	173.48	7.35
1	-4	3	186.96	5.56
1	4	-3	187.44	8.36
-1	-4	3	181.48	5.37
1	4	4	37.75	4.34
-1	4	-4	51.28	5.10
-1	4	-4	45.88	5.18

1	4	-4	31.83	5.21
1	-4	4	39.34	3.72
-1	-4	4	38.38	3.68
1	4	5	48.34	4.52
-1	4	-5	45.24	5.01
1	4	-5	53.88	5.06
-1	4	-5	50.98	5.41
1	4	-5	54.92	5.75
1	-4	5	41.06	3.56
-1	-4	5	46.67	3.72
-1	4	-6	85.84	5.58
1	4	-6	92.42	5.67
-1	4	-6	78.40	5.87
1	4	-6	78.57	6.12
1	-4	6	77.10	4.35
-1	-4	6	79.58	4.20
-1	-4	7	44.15	3.74
-1	4	-7	60.30	5.26
1	4	-7	57.51	5.06
-1	4	-7	44.56	4.93
1	4	-7	39.56	5.34
1	-4	7	48.91	3.58
1	-4	8	0.34	2.71
-1	-4	8	5.39	2.75
-1	4	-8	-0.55	3.92
1	4	-8	1.28	3.90
1	-4	9	0.81	2.34
-1	-4	9	-1.38	2.59
1	4	-9	5.48	3.77
2	4	0	-4.44	5.87
-2	-4	0	-1.66	2.40
2	-4	0	2.51	3.35
2	4	0	0.26	4.17
2	4	-1	5.48	6.03
2	4	1	2.57	5.22
-2	-4	1	2.47	2.71
2	-4	-1	0.74	3.17
2	4	-1	-4.76	4.58
2	-4	1	1.24	3.28
2	4	-2	53.09	5.47
2	-4	-2	52.47	3.89
2	-4	2	51.19	4.05
2	4	-2	53.38	7.05
-2	-4	2	52.09	3.62
2	4	2	45.74	5.36
2	4	3	102.02	5.70
-2	4	-3	100.22	5.65
-2	4	-3	111.24	6.07
2	4	-3	109.16	6.33
2	-4	-3	96.78	4.44
2	-4	3	113.68	4.80
2	4	-3	107.97	7.46
-2	-4	3	110.13	4.47
2	4	-4	9.65	6.01
2	-4	4	2.62	3.21
2	4	4	5.81	4.09
-2	-4	4	6.37	3.11
-2	4	-4	3.60	4.46
-2	4	-4	-2.26	4.21
2	4	-4	0.74	4.72
-2	-4	5	131.32	5.04
2	4	5	112.09	5.65
-2	4	-5	124.90	6.05
-2	4	-5	116.22	6.06

2	4	-5	119.09	6.78
2	-4	5	110.50	4.66
-2	-4	6	73.24	4.15
-2	4	-6	66.39	5.27
-2	4	-6	66.33	5.23
2	4	-6	74.50	5.92
2	-4	6	61.04	3.84
2	4	-7	-2.06	3.78
2	-4	7	2.11	2.76
-2	4	-7	2.09	3.96
2	4	-7	-3.02	4.46
-2	4	-7	-6.09	4.11
-2	-4	8	19.13	3.22
2	-4	8	13.45	2.63
-2	4	-8	15.43	4.14
3	4	0	1.21	5.33
3	-4	0	-0.82	3.31
3	4	0	-1.68	4.19
3	4	-1	215.02	7.43
3	-4	-1	214.50	5.88
3	-4	1	213.98	5.90
3	4	-1	193.29	8.01
3	4	1	213.05	8.01
-3	-4	1	212.78	5.01
-3	4	-2	224.32	6.66
3	4	-2	208.41	7.72
3	-4	-2	211.56	6.01
3	-4	2	212.67	5.84
3	4	-2	213.39	8.35
-3	-4	2	231.56	5.53
3	4	2	224.80	7.78
-3	-4	3	18.92	3.34
3	4	3	28.54	4.96
3	4	-3	27.77	6.08
3	4	-3	20.31	5.35
3	-4	-3	21.09	3.72
-3	4	-3	16.23	4.08
3	-4	3	23.87	3.67
3	-4	4	3.22	3.17
3	4	-4	3.26	5.87
3	4	4	-1.43	4.12
3	-4	-4	-1.55	3.34
-3	-4	4	2.26	3.10
-3	4	-4	-5.27	4.11
3	4	-4	3.04	4.80
3	-4	5	-1.87	3.15
3	4	5	1.74	3.91
-3	-4	5	5.05	3.04
-3	4	-5	-2.21	4.05
3	4	-6	2.22	4.85
3	4	6	8.79	4.03
3	-4	6	2.51	2.78
-3	-4	6	7.18	3.03
-3	4	-6	0.77	4.00
-3	4	-6	3.97	3.96
3	4	-6	1.75	4.71
3	-4	7	5.07	2.65
-3	4	-7	19.00	4.16
-3	-4	7	7.47	2.97
-3	4	-7	16.08	4.12
3	4	-7	9.76	4.82
3	-4	8	5.65	2.31
-3	4	-8	-0.01	3.77
-3	-4	8	0.07	2.85

4	4	0	2.21	4.89
4	-4	0	5.55	3.45
4	4	0	6.90	4.44
-4	-4	1	40.42	3.06
4	4	-1	43.20	5.22
4	-4	-1	38.71	3.99
4	-4	1	49.84	4.02
4	4	-1	46.61	5.93
4	4	1	40.64	5.42
-4	-4	2	93.94	3.99
-4	4	-2	80.64	4.80
4	4	-2	72.41	5.77
4	-4	-2	78.18	4.59
4	-4	2	74.27	4.23
4	4	-2	80.12	6.12
4	4	2	78.43	6.11
4	4	-3	9.76	5.31
4	4	3	10.04	4.44
4	-4	-3	9.48	3.50
-4	-4	3	3.12	2.84
-4	4	-3	5.70	3.75
4	4	-3	10.93	4.87
4	-4	3	12.04	3.09
4	4	4	47.14	4.92
-4	-4	4	45.77	3.88
-4	4	-4	39.16	4.44
4	-4	-4	40.66	4.13
4	4	-4	54.81	5.46
4	-4	4	42.17	3.64
4	4	-4	43.24	5.99
4	4	-5	1.40	5.18
4	-4	5	1.00	2.84
4	4	5	2.99	3.93
4	-4	-5	-5.38	3.50
-4	4	-5	-0.41	3.65
-4	-4	5	-1.35	2.96
4	4	-5	-0.39	4.76
4	4	6	72.22	5.03
-4	-4	6	63.60	4.10
-4	4	-6	75.78	4.99
4	4	-6	77.37	5.88
4	-4	6	89.04	4.02
4	4	-6	88.42	6.33
-4	-4	7	15.89	3.23
-4	4	-7	12.70	3.82
4	4	-7	23.89	4.98
4	-4	7	21.53	2.66
5	4	0	0.15	4.42
5	-4	0	2.74	3.21
5	4	0	-0.35	4.28
5	4	-1	7.23	4.55
5	4	1	14.93	4.74
5	-4	-1	14.74	3.63
5	-4	1	19.37	3.47
5	4	-1	10.30	4.62
-5	-4	1	12.82	2.59
-5	-4	2	20.77	2.96
5	4	2	22.25	4.65
-5	4	-2	29.38	3.97
5	-4	-2	28.13	3.87
5	-4	2	24.81	3.28
5	4	-2	22.21	5.01
5	4	-2	30.37	5.14
-5	-4	3	9.70	2.99

5	4	-3	6.97	4.76
5	4	3	7.68	4.32
5	-4	-3	3.69	3.49
-5	4	-3	10.20	3.85
5	4	-3	5.34	4.82
5	-4	3	7.10	3.16
5	4	4	142.01	6.09
-5	-4	4	152.32	5.23
-5	4	-4	141.19	5.70
5	-4	-4	141.96	5.51
5	4	-4	149.79	7.19
5	-4	4	140.00	4.73
5	4	-4	147.91	6.95
5	-4	5	3.60	2.71
5	4	-5	5.07	4.68
5	4	5	-0.48	3.87
5	-4	-5	-0.33	3.36
-5	4	-5	3.37	3.50
5	4	-5	-1.91	4.55
-5	-4	5	0.21	3.04
5	4	-6	-1.86	4.47
5	4	6	1.23	3.74
5	-4	6	3.27	2.36
5	-4	-6	-2.65	3.22
-5	4	-6	-0.70	3.42
-5	-4	6	0.88	2.92
5	-4	7	-1.78	2.11
6	-4	0	9.32	3.21
6	4	0	19.66	4.55
6	4	1	56.67	5.04
6	-4	-1	54.77	4.02
6	-4	1	59.00	3.82
6	4	-1	47.69	4.78
-6	-4	2	3.66	2.60
6	4	-2	-0.04	3.97
6	4	2	-5.58	4.06
6	-4	-2	-0.08	3.29
-6	4	-2	-5.07	3.45
6	-4	2	4.43	2.91
6	4	-2	1.47	4.60
6	4	3	25.06	4.19
-6	-4	3	25.48	3.22
-6	4	-3	32.42	4.20
6	-4	-3	40.38	4.10
6	4	-3	24.21	5.16
6	-4	3	29.97	3.22
6	4	-3	28.47	4.64
-6	-4	4	5.01	2.90
6	4	-4	8.10	4.40
6	4	4	3.38	3.75
6	-4	-4	7.85	3.44
-6	4	-4	3.94	3.61
6	4	-4	2.84	4.62
6	-4	4	4.81	2.55
6	-4	5	0.65	2.29
6	4	-5	-7.03	4.31
-6	-4	5	-4.16	2.83
6	4	5	0.23	3.64
6	-4	-5	-3.07	2.98
-6	4	-5	4.53	3.50
7	4	0	2.44	3.86
7	-4	0	-0.04	2.84
7	4	1	19.50	3.98
7	4	-1	27.90	4.52

7	-4	-1	21.47	3.26
7	-4	1	30.56	3.17
7	4	-1	23.95	4.11
7	4	2	46.37	4.49
-7	-4	2	44.60	3.50
-7	4	-2	47.44	4.18
7	-4	-2	45.43	3.85
7	-4	2	55.32	3.49
7	4	-2	35.57	4.39
7	4	-3	6.93	3.64
7	4	3	8.43	3.56
-7	-4	3	3.48	2.57
7	-4	-3	8.99	3.24
-7	4	-3	15.95	3.68
7	-4	3	15.74	2.77
7	4	-4	6.07	3.65
-7	-4	4	15.33	2.99
-7	4	-4	14.09	3.75
0	5	0	-2.53	4.62
0	-5	1	-0.10	2.70
0	5	-1	-6.87	5.80
0	5	-2	-1.80	5.66
0	-5	2	-3.80	2.77
0	5	-3	107.52	5.66
0	-5	3	118.98	4.36
0	5	-3	127.77	7.53
0	5	-4	-4.44	5.45
0	-5	4	-2.80	2.77
0	5	-4	-3.77	4.19
0	5	-5	83.28	5.41
0	5	-5	80.75	6.48
0	-5	5	79.94	3.92
0	-5	6	-0.75	2.41
0	-5	7	23.80	3.00
1	5	0	-2.43	5.01
1	-5	0	4.79	2.63
1	5	-1	3.29	5.74
1	5	1	3.75	4.58
-1	-5	1	4.40	2.50
1	-5	1	5.79	2.81
-1	5	-2	106.50	5.67
1	5	-2	111.05	5.81
-1	-5	2	109.90	4.32
1	5	-2	106.67	7.00
1	5	2	101.14	5.47
1	5	-3	44.18	4.75
-1	5	-3	31.88	4.34
1	-5	3	40.57	3.34
-1	5	-3	37.88	5.55
1	5	-3	38.60	6.57
-1	-5	3	42.65	3.47
-1	5	-4	-2.61	4.80
1	5	-4	-7.82	6.23
1	-5	4	3.32	2.74
-1	-5	4	2.38	2.61
-1	5	-4	3.43	3.97
1	5	-4	-0.28	4.08
1	5	-5	0.10	5.82
1	-5	5	-0.44	2.54
-1	-5	5	-2.22	2.58
-1	5	-5	-5.96	4.29
1	5	-5	-0.21	4.21
1	5	-6	10.28	5.40
1	-5	6	8.81	2.65

-1	-5	6	5.00	2.65
2	-5	0	124.28	4.46
2	5	0	103.61	6.56
2	-5	-1	92.73	3.89
2	-5	1	107.63	4.25
2	5	-1	95.72	6.87
2	5	1	99.96	6.26
2	5	-2	16.59	5.68
2	5	2	20.85	4.78
-2	5	-2	20.12	4.00
2	5	-2	20.40	4.27
-2	-5	2	11.04	2.72
2	-5	2	12.54	2.88
2	-5	3	13.87	2.99
2	5	-3	26.43	6.03
2	5	3	20.00	4.14
-2	5	-3	16.08	4.01
2	5	-3	16.40	4.40
-2	-5	3	23.55	3.05
2	5	-4	4.25	5.95
2	-5	4	7.96	2.73
2	5	4	-1.53	3.69
-2	-5	4	6.80	2.77
-2	5	-4	-2.67	3.67
2	5	-4	-2.78	4.07
-2	-5	5	25.34	3.10
-2	5	-5	30.14	4.34
2	5	-5	22.01	4.60
2	-5	5	29.20	3.07
2	5	-6	10.22	5.70
2	-5	6	6.13	2.69
-2	-5	6	2.98	2.61
3	5	0	2.15	4.93
3	-5	0	-0.21	2.65
3	5	-1	10.23	5.06
3	5	1	21.25	5.01
3	-5	-1	25.46	3.11
3	-5	1	14.90	3.01
3	5	-2	-1.25	5.28
3	5	2	-6.00	4.53
-3	-5	2	4.83	2.50
3	-5	-2	4.01	2.64
-3	5	-2	1.39	3.51
3	5	-2	-3.79	4.04
3	-5	2	-0.02	2.67
3	5	-3	-5.13	5.55
3	5	3	1.50	4.21
-3	-5	3	1.44	2.63
-3	5	-3	5.97	3.60
3	5	-3	12.30	4.35
3	-5	3	4.68	2.78
-3	-5	4	103.40	4.37
3	5	4	107.67	5.46
-3	5	-4	98.80	5.21
3	5	-4	107.89	5.92
3	-5	4	103.30	4.23
3	5	-4	100.53	7.23
-3	-5	5	20.41	3.03
3	5	-5	15.14	6.03
3	5	5	17.71	3.75
3	-5	5	24.48	2.94
3	5	-6	5.94	5.61
3	-5	6	5.37	2.52
-3	-5	6	6.17	2.59

4	5	0	0.13	4.55
4	-5	0	1.66	2.60
4	5	-1	5.58	4.55
4	5	1	13.08	4.61
4	-5	-1	18.23	2.93
4	-5	1	11.51	2.91
4	5	-2	10.85	4.72
4	5	2	14.54	4.52
4	-5	-2	13.78	2.95
4	-5	2	13.47	2.87
4	5	-3	3.66	5.01
4	5	3	1.35	4.07
4	-5	-3	3.63	2.57
-4	-5	3	7.17	2.50
-4	5	-3	10.72	3.59
4	5	-3	5.04	4.04
4	-5	3	8.29	2.63
4	-5	4	10.28	2.69
4	5	-4	0.21	5.17
4	5	4	4.73	3.87
-4	-5	4	-0.33	2.52
4	5	-5	5.92	5.17
4	-5	5	10.81	2.60
4	5	5	13.20	3.77
-4	-5	5	10.84	2.89
5	5	0	-0.97	4.09
5	-5	0	1.25	2.58
5	5	1	104.14	5.76
5	-5	-1	95.52	4.18
5	-5	1	96.17	4.17
5	5	-1	88.00	5.33
5	5	-2	1.47	4.16
5	5	2	4.12	4.08
5	-5	-2	2.09	2.82
5	-5	2	0.34	2.49
-5	-5	3	16.93	2.68
5	5	-3	11.25	4.36
5	5	3	13.17	3.95
5	-5	-3	10.11	2.88
5	-5	3	15.17	2.70
-5	-5	4	18.36	2.90
5	-5	-4	6.97	2.85
6	5	0	-4.11	3.96
6	-5	0	4.95	2.67
6	-5	-1	1.88	2.57
0	-6	2	-3.83	2.19
0	-6	3	2.72	2.50
1	-6	1	2.41	2.13
1	-6	2	5.30	2.35
1	-6	3	31.56	2.88
-1	-6	3	35.67	2.92
2	6	0	12.41	4.45
2	-6	1	3.78	2.23
2	6	-1	-5.30	4.76
2	-6	2	7.26	2.43
2	6	-2	1.03	4.55
-2	-6	3	23.31	2.64
3	-6	1	43.06	3.00
0	0	0	0.00	0.00

```
_computing_structure_solution     'SHELXT 2014/5 (Sheldrick, 2014)'
;
_shelx_hkl_checksum               23729
_olex2_submission_special_instructions 'No special instructions were received'
```
