# Supplementary material for: Allabogdanite, the high-pressure polymorph of (Fe,Ni)2P, a stishovite-grade indicator of impact processes in the Fe–Ni–P system
Source: Sci Rep. 2019 Jan 31;9:1047. doi: 10.1038/s41598-018-37795-x (PMC6355872; doi:10.1038/s41598-018-37795-x)
Supplement: Supplementary file 2 — Supplementary Information [file 41598_2018_37795_MOESM2_ESM.pdf]

Supplementary Table 1. Crystal parameters, data collection and structure refinement details for allabogdanite from the Santa Catharina meteorite.

**Parameter**	**Value**
Formula	$(Fe_{1.33}Ni_{0.67})_{2.00}P$
Crystal system	Orthorhombic
Space group	*Pnma*
a (Å)	5.7332(7)
b (Å)	3.5413(6)
c (Å)	6.6682(10)
V (Å^3)	135.38(3)
Z	4
D_{calc} (g cm^{-3})	7.09
Crystal size (mm)	0.005 × 0.01 × 0.02
Radiation	Mo*K*α (λ = 0.71073 Å)
Temperature (*K*)	293
2*Θ* range (°)	4.0–52.00
Total reflections collected	1075
Unique reflections	152
Unique observed reflections [$I \geq 2\sigma(I)$]	136
$R_{int.}$	0.0164
R_{σ}	0.0076
h, k, l range	–7→7, –4→4, –8→8
R_1 [$F \geq 4\sigma(F)$]	0.0426
R_1 (all data)	0.0455
wR_2	0.1051
S = GoF	1.206

Supplementary Table 2. Fractional atomic coordinates and isotropic displacement parameters (Å^2) for allabogdanite from the Santa Catharina meteorite

Site	*x*	*y*	*z*	U_{eq}
*M*1*	0.3583(2)	¼	0.9361(2)	0.0279(6)
*M*2*	0.4692(2)	¼	0.3329(2)	0.0334(7)
P	0.2453(4)	¼	0.6227(5)	0.0302(8)

*Occupancy of *M*1 and *M*2 sites: $Fe_{0.67}Ni_{0.33}$

Supplementary Table 3. Anisotropic displacement parameters (Å^2) for allabogdanite from the Santa Catharina meteorite

Site	U_{11}	U_{22}	U_{33}	U_{23}	U_{13}	U_{12}
*M*1	0.0293(8)	0.0269(9)	0.0273(9)	0	-0.0002(5)	0
*M*2	0.0360(10)	0.0319(10)	0.0323(10)	0	-0.0003(5)	0
P	0.0319(12)	0.0283(14)	0.0306(15)	0	0.0011(10)	0

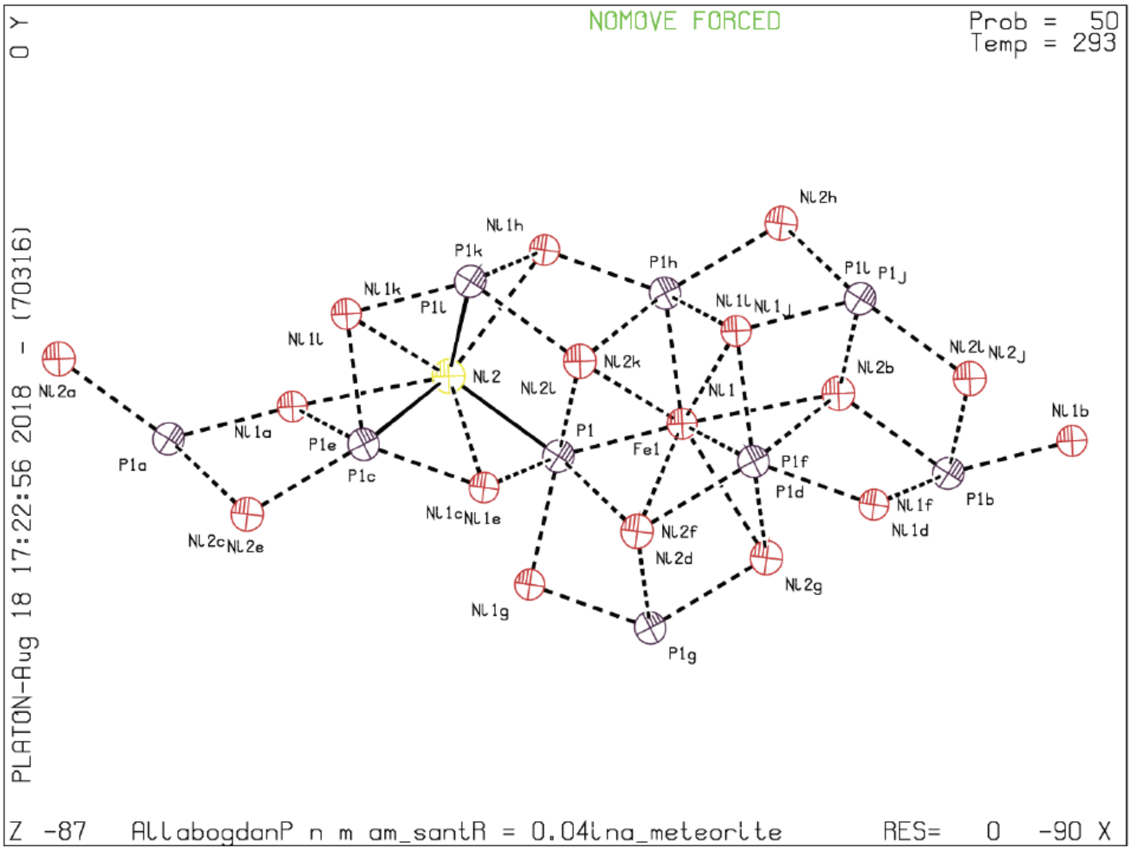

Supplementary Figure 1. An ORTEP view of the crystal structure of allabogdanite from the Santa Catharina meteorite.
